# Thermo-Mechanical Compatibility of Viscoelastic Mortars for Stone Repair

**DOI:** 10.3390/ma9010056

**Published:** 2016-01-18

**Authors:** Thibault Demoulin, George W. Scherer, Fred Girardet, Robert J. Flatt

**Affiliations:** 1 Physical Chemistry of Building Materials, Institute for Building Materials, HIF, ETH Zurich, Zurich 8093, Switzerland; tdemoulin@ethz.ch; 2 Department of Civil and Environmental Engineering, Eng. Quad. E-319, Princeton University, Princeton, NJ 08544, USA; scherer@princeton.edu; 3 RINO Sarl, Blonay 1807, Switzerland; fred.girardet@bluewin.ch

**Keywords:** reprofiling, filling, patch, acrylic polymer, repair, artificial stone, mortar, thermal stress, viscoelasticity, on-site measurement

## Abstract

The magnitude of the thermal stresses that originate in an acrylic-based repair material used for the reprofiling of natural sandstone is analyzed. This kind of artificial stone was developed in the late 1970s for its peculiar property of reversibility in an organic solvent. However, it displays a high thermal expansion coefficient, which can be a matter of concern for the durability either of the repair or of the underlying original stone. To evaluate this risk we propose an analytical solution that considers the viscoelasticity of the repair layer. The temperature profile used in the numerical evaluation has been measured in a church where artificial stone has been used in a recent restoration campaign. The viscoelasticity of the artificial stone has been characterized by stress relaxation experiments. The numerical analysis shows that the relaxation time of the repair mortar, originating from a low $T_{g}$, allows relief of most of the thermal stresses. It explains the good durability of this particular repair material, as observed by the practitioners, and provides a solid scientific basis for considering that the problem of thermal expansion mismatch is not an issue for this type of stone under any possible conditions of natural exposure.

## 1. Introduction

**Figure 1:**
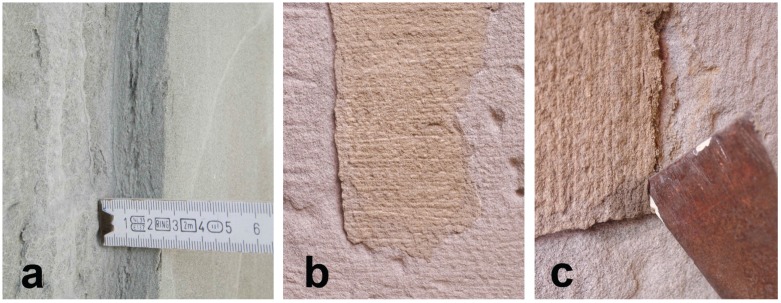
(**a**) Common dimension of a flake in a historical building molasse sandstone; (**b**) Old acrylic-based mortar used in Lausanne, after more than 30 years; (**c**) Reversibility of the old acrylic-based mortar.

Many dimension stones used for the construction of historical buildings show, after a certain time, superficial degradations in the first centimeters that do however not affect the stone below this depth. That is the case, for instance, during the formation of scales in a sandstone during a spalling process. These alterations are formed parallel to the outer surface and are independant of the stone bedding orientation, suggesting that a combination of transport properties and environmental exposure causes the stress from a degradation process to reach critical levels only at a certain depth. An example of such alteration is presented in Figure 1a.

When decisions are made to restore these stones, the question is how to remediate these alterations: should the degraded part of the stone be replaced by another stone, or by an appropriate mortar? Since the alteration is generally superficial, a reinstatement of natural stones would imply a removal of potentially sound original material to a depth of at least 10 cm to ensure a good placement [1], while the use of a plastic mortar that can be substituted for the lost parts would result in a minimal loss of historical material and, in addition, extend its lifetime. The latter practice is called “reprofiling” or “filling”, while the piece itself is called “plastic repair” [2], “piecing-in” [1], “fill” or “patch”. This strategy complies with modern building conservation principles that favour a minimal intervention and the retaining of as much historical material as possible [2]. It is one of the reasons that explains the increasing use of repair mortar in conservation, as pointed out by Torney in Scotland [2], and the increasing research undertaken, in both the philosophical side of the repair [3,4,5] and the material science side, with the development of compatibility criteria, upon which Isebaert wrote a recent review [6], as well as new mortar formulations [7] sometimes based on polymers [8].

The present work was initiated by the curiosity and questioning of stone carvers who used an acrylic-based mortar for the repair of calcareous sandstones in the late seventies in Switzerland. This mortar was developed at that time by Professor Furlan’s team in the Ecole Polytechnique Fédérale de Lausanne, and applied in the townhall of Lausanne, in locations protected from the direct sun but washed weekly by a high-pressure water hose to clean the remnants of the market. More than thirty years later, the mortar is in most of the locations still in place, and the repairs have been judged durable and successful by the stone carvers from the point of view of the integrity of the natural stone it aimed to repair, as shown in Figure 1b. The stone carvers thus proposed this mortar for the restoration of the Catholic Church of Notre-Dame de Vevey, Vevey, Switzerland, where it was used in 2011. However, owing to the very different natures of the original stone and the repair mortar, a more comprehensive understanding of the interactions between them would provide the foundation to decide in which situations this material could be beneficial or not.

The development of an acrylic-based repair mortar by Professor Furlan’s team originated in the confluence of three factors: the particular mode of alteration of the local stone, the resurgence of interest towards repair mortars due to the spreading of the minimal-intervention approach, and the increasing use of polymers in conservation.

Indeed, many of the Swiss historical buildings have been erected with the soft stone present in the Swiss Plateau, a sandstone called molasse, mainly composed of quartz and felspars cemented by calcite and clays [9]. This stone is sensitive to wetting and drying cycles that commonly lead to granular desintegration and spalling of flakes of 0.5 to 3 cm [10], but the stone is often in good conditions above this limit, as illustrated by Figure 1a.

Eventually, the properties of reversibility, transparency and stability generally attributed to acrylic polymers [1,11] oriented the team towards devising a mortar with an acrylic binder. These properties contributed to the large use of acrylic polymers in the conservation of heritage materials. Since the early 1930’ s, where they were used as picture varnishes [12], they have been applied on glass pigments, paper, silver, iron, wood and stone [13]. In the particular field of stone conservation, they have mainly been used, alone or mixed with other polymers, as consolidants or protective agents [1,11]. The search for better and more stable polymers led to the development of many formulations; in a recent handbook Princi listed 31 different commercial acrylic resins applied to stone conservation [11]. However, even though they are often used for the conservation of stone objects, there are no detailed records in the scientific literature. Moreover, to the best of the authors’ knowledge, there are no reports of their use as the sole binder in a repair mortar, to the exception of Kemp who describes the repair of marble using artificial stones based on Paraloid [14].

The mortar that is the subject of this paper is made by mixing a stone powder with a dispersion of methacrylic ester-acrylic ester copolymer. The hardening occurs through the drying of the dispersion and the subsequent coalescence of the polymer particles, that ultimately bind the grains together. Due do its slow drying time, this artificial stone is better indicated for repairs no thicker than a couple of centimeters, while lateral dimensions can reach few tens of centimeters, which overall agrees well with the maximum dimensions generally accepted for a repair. In the hardened state, the polymer represents 10 % of the weight of the stone powder and is the only binder between the grains. This has several consequences : because it is transparent, the color of the artificial stone is the color of the stone powder, making it easy to match the color of the natural stone; because the polymer can be dissolved in an organic solvent, the mortar is reversible and can be removed without breaking the original stone substrate when, or if, time has come for another repair. It has to be noted that the reversibility is still effective after more than thirty years of use in the townhall of Lausanne, as can be seen in Figure 1c. The stone repaired in this study is a calcareous sandstone but the repair material could potentially be used on any stone that would benefit from a small-size repair and that does not display any visible pattern or layering. Another interesting feature lies in its ability to be worked like the sandstone due to its similar stiffness, thus making the integration of the repair possible by the normal tools of a stone carver.

However, repair with an acrylic-based mortar does not correspond to the "like-for-like" principle of building conservation, that would favour using a similar material for a repair [5]. The sandstone is indeed a soft elastic material, while the mortar displays the physical properties of the acrylic resin, among which viscoelasticity, namely time and temperature-dependent physical properties, and a thermal expansion coefficient more than twice that of the molasse sandstone. This raises questions about the magnitude of the thermal stresses induced in the two materials. Indeed, on one hand, on repairs exposed outdoors and subject to temperature variations, the thermal expansion mismatch could lead to high thermal stresses in the two materials. On the other hand, the viscoelastic character of the repair mortar may attenuate the development of these thermal stresses, so that they should not only depend on the temperature difference, but also on the rate at which this temperature difference develops. The magnitude of the thermal stresses is thus dictated by the site exposure and developing a general treatment to evaluate their importance represents an important objective for providing more confidence in the use of such repair materials from case to case.

The aim of this work is thus to calculate the thermal stresses arising in the two materials at heating and cooling rates representative of field exposure. We present the general approach for this and illustrate its use with site specific numerical examples. For this, we use temperature variations that have been recorded over a year at different depths in a wall where the repair mortar has recently been applied, in the Catholic Church of Notre-Dame de Vevey. The physical properties of the materials, namely the thermal expansion coefficient, the elastic moduli, and the viscoelastic modulus of the artificial stone have been measured in the laboratory. The numerical evaluation is achieved through the development of an analytical solution presented in the next section.

## 2. Mechanical Analysis of the Repair

**Figure 2:**
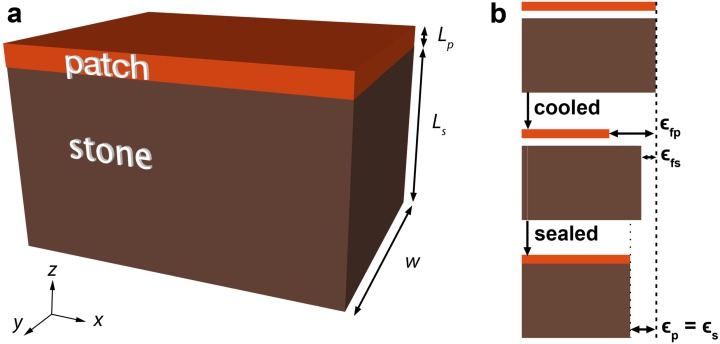
(**a**) Schematic of the patch layer on top of the stone substrate; (**b**) Illustration of the thermal expansion mismatch between the two materials.

The simplest case of reprofiling can be seen as a bilayer composite material made of a thin mortar layer (“patch”) on a thick stone substrate. If we extract a part of the composite from the wall, it would look like the scheme of Figure 2a.

When two materials are bound together, if they have dissimilar expansion properties, mismatch forces arise at the interface and internal stresses build up. That is the case for thermal expansion mismatch, as is illustrated in Figure 2b. Consider two plates of repair mortar and natural stone of equal length; after cooling, the patch contracts by $\epsilon_{f,p}$ and the stone by $\epsilon_{f,s}$, respectively the free strain of the patch and the stone. However, if they were connected, they would have contracted by an amount $\epsilon_{p}=\epsilon_{s}$ intermediate between $\epsilon_{f,p}$ and $\epsilon_{f,s}$. This imposed strain is, as we shall see, dictated by the elastic moduli, the Poisson’s ratios and the relative thicknesses of both materials, and leads to the creation of internal stresses. But, since the repair material has a polymeric nature and exhibits a viscoelastic behaviour, the stresses developed in it might be relaxed. Consequently the stresses affecting the natural stone might decrease, depending on the temperature of the material and on the heating or cooling rate. This section develops an analytical description to quantify whether this is the case or not, and if so under which conditions of temperature. This analysis follows the steps and the notation developed in the analysis of the sandwich seal [15]. The stone is considered linear elastic and the acrylic-based repair material is considered viscoelastic.

Let us define a Cartesian coordinate system where the x and y-axis are parallel to the layers. Since the composite is not constrained in the z-direction, $\sigma_{z}$ = 0 ; and due to the symmetry of the composite, $\sigma_{x}=\sigma_{y}$ in the two layers. In all the subsequent equations, the subscripts *p* and *s* stand for patch and stone. If the materials are considered isotropic, and the stress in the z-direction is zero, the constitutive equations connecting stress and strain can be written:

(1) $$\epsilon_{p}=\epsilon_{f,p}+\frac{1}{E_{p}}\left(\sigma_{p}-\nu_{p}\sigma_{p}\right)$$

and

(2) $$\epsilon_{s}=\epsilon_{f,s}+\frac{1}{E_{s}}\left(\sigma_{s}-\nu_{s}\sigma_{s}\right)$$

where $\epsilon_{f}$ stands for the free strain of the material; *E* for the elastic modulus; and *ν* for the Poisson’s ratio. Due to the bond between the two materials, the layers have strained equally in the central region of the repair, so $\epsilon_{p}$ = $\epsilon_{s}$. Thus,

(3) $$\epsilon_{f,p}+\frac{1-\nu_{p}}{E_{p}}\sigma_{p}=\epsilon_{f,s}+\frac{1-\nu_{s}}{E_{s}}\sigma_{s}$$

Another equation to define $\sigma_{s}$ can be obtained by considering the edge of the composite, where the net force in the isolated sample must be zero. Let us call the thicknesses of the layers $L_{p}$ for the patch and $L_{s}$ for the stone substrate; they share the same width *w*, and thus we have:

(4) $$L_{s}w\sigma_{s}+L_{p}w\sigma_{p}=0$$

or

(5) $$\sigma_{s}=-\frac{L_{p}}{L_{s}}\sigma_{p}$$

Combining Equation (3) and Equation (5), we find:

(6) $$\sigma_{p}=\frac{E_{p}\left(\epsilon_{f,s}-\epsilon_{f,p}\right)}{\left(1-\nu_{p}\right)\left(1+n\right)}$$

with *n* the stiffness ratio defined as:

(7) $$n=\frac{L_{p}}{L_{s}}\frac{E_{p}}{1-\nu_{p}}\frac{1-\nu_{s}}{E_{s}}$$

In a typical reprofiling the thickness of the patch is very small compared to the thickness of the stone substrate, on the order of 1/20. If we consider $L_{p}$*<<*$L_{s}$, then *n* → 0 and the stress is maximal in the repair layer; on the other side, the repair layer does not exert significant stress on the stone. The maximal elastic stress exerted by the stone on the repair layer is then expressed by:

(8) $$\sigma_{p}=\left(\frac{E_{p}}{1-\nu_{p}}\right)\left(\epsilon_{f,s}-\epsilon_{f,p}\right)$$

The free strain $\epsilon_{f}$ is the unconstrained change of dimension of the material. It is given by:

(9) $$\epsilon_{f,s}=\int_{T_{0,s}}^{T_{s}}\alpha_{s}\;dT$$

and

(10) $$\epsilon_{f,p}=\int_{T_{0,p}}^{T_{p}}\alpha_{p}\;dT$$

with $\alpha_{s}$ and $\alpha_{p}$ the coefficient of thermal expansion of the stone and the patch; and $dT_{s}$ and $dT_{p}$ the infinitesimal temperature variation of the stone and the patch, respectively. Since the coefficients of thermal expansion are taken independent of the temperature, the elastic thermal stress can be written:

(11) $$\sigma_{p}=\left(\frac{E_{p}}{1-\nu_{p}}\right)\left(\alpha_{s}\Delta T_{s}-\alpha_{p}\Delta T_{p}\right)$$

with ΔT the temperature differences. This equation thus describes the maximal stresses that can appear in the repair material. The viscoelastic solution, according to the correspondence principle, is found by substituting the elastic quantities by their appropriate transformed quantities in the Laplace domain [15,16,17,18]. When using a dash superscript to indicate the Laplace transform, the Equation (8) in the Laplace domain is written:

(12) $${\bar{\sigma }}_{p}=\left(\frac{M}{1-N}\right)\left({\bar{\epsilon }}_{f,s}-{\bar{\epsilon }}_{f,p}\right)$$

where the elastic modulus is transformed into an apparent modulus *M* and the Poisson’s ratio into an apparent Poisson’s ratio *N;* here, the relaxation of Poisson’s ratio is ignored. The apparent modulus *M* is related to the elastic modulus *E* through the transform of the relaxation function $\bar{\psi }$. In case of a uniaxial stress, M is written:

(13) $$M=E_{p}p\bar{\psi }$$

where $E_{p}$ is the instantaneous elastic modulus of the patch and *p* is the Laplace transform parameter. It follows that

(14) $${\bar{\sigma }}_{p}=\left(\frac{E_{p}}{1-\nu_{p}}\right)p\left({\bar{\epsilon }}_{f,s}-{\bar{\epsilon }}_{f,p}\right)\bar{\psi }$$

The solution in the Laplace domain has to be inverted to find the solution in the time domain. The stress in the patch becomes:

(15) $$\sigma_{p}=\frac{E_{p}}{1-\nu_{p}}\;\int_{0}^{\xi }\psi \left[\xi -\xi^{\prime }\right]\frac{d\left(\epsilon_{f,s}-\epsilon_{f,p}\right)}{d\xi^{\prime }}d\xi^{\prime }$$

where *ξ* is the reduced time defined as

(16) $$\xi =\int_{0}^{t}\frac{dt^{\prime }}{\tau }$$

and thus

(17) $$d\xi =\frac{dt}{\tau }$$

in which the relaxation time of the artificial stone is *τ*. As is confirmed in the experimental section, the relaxation function has the form of a stretched exponential:

(18) $$\psi \left[t\right]=\psi_{0}+\left(1-\psi_{0}\right)\exp\left(-{\left(t/\tau \right)}^{\beta }\right)$$

which, together with Equation (17), when substituted into Equation (15), gives:

(19) $$\sigma_{p}=\frac{E_{p}}{1-\nu_{p}}\left(\psi_{0}\left(\epsilon_{f,s}-\epsilon_{f,p}\right)+\left(1-\psi_{0}\right)\int_{0}^{\xi }\exp\left(-\left(\int_{t^{\prime }}^{t}\frac{dt^{\prime \prime }}{\tau [T_{p}]}\right){}^{\beta }\right)\frac{d\left(\epsilon_{f,s}-\epsilon_{f,p}\right)}{d\xi^{\prime }}d\xi^{\prime }\right)$$

In Equation (19) the relaxation time is written as $\tau [T_{p}]$ since it is temperature dependent. From the stress relaxation experiments at different temperatures, the artificial stone shows a thermorheologically simple behaviour that can be described by an Arrhenius law (see results in corresponding section). The dependence of the relaxation time with the temperature is thus written :

(20) $$\tau =\tau_{0}\exp\left(\frac{Q}{T_{p}}\right)$$

and can be incorporated in Equation (19):

(21) $$\sigma_{p}=\frac{E_{p}}{1-\nu_{p}}\left(\psi_{0}\left(\epsilon_{f,s}-\epsilon_{f,p}\right)+\left(1-\psi_{0}\right)\int_{0}^{\xi }\exp\left(-\left(\int_{t^{\prime }}^{t}\frac{dt^{\prime \prime }}{\tau_{0}\exp\left(\frac{Q}{T_{p}}\right)}\right){}^{\beta }\right)\frac{d\left(\epsilon_{f,s}-\epsilon_{f,p}\right)}{d\xi^{\prime }}d\xi^{\prime }\right)$$

Since the temperatures of the two materials are different, then

(22) $$\frac{d\left(\epsilon_{f,s}-\epsilon_{f,p}\right)}{d\xi^{\prime }}=\alpha_{s}\;\frac{dT_{s}}{d\xi^{\prime }}-\alpha_{p}\;\frac{dT_{p}}{d\xi^{\prime }}$$

The derivative in *ξ* can be transformed in derivative in *t* thanks to Equation (17). By dividing by τ, the previous equation thus becomes

(23) $$\frac{d\left(\epsilon_{f,s}-\epsilon_{f,p}\right)}{dt^{\prime }}=\alpha_{s}\;\frac{dT_{s}}{dt^{\prime }}-\alpha_{p}\;\frac{dT_{p}}{dt^{\prime }}$$

and we need to distinguish the integrals for the two materials in Equation (21). They are written:

(24) $$\Delta_{s}=\int_{0}^{t}\exp\left(-\left(\int_{t^{\prime }}^{t}\frac{dt^{{}^{\prime \prime }}}{\tau_{0}\exp\left(\frac{Q}{T_{p}\left[t^{\prime \prime }\right]}\right)}\right){}^{\beta }\right)\frac{dT_{s}}{dt^{\prime }}\;dt$$

and

(25) $$\Delta_{p}=\int_{0}^{t}\exp\left(-\left(\int_{t^{\prime }}^{t}\frac{dt^{{}^{\prime \prime }}}{\tau_{0}\exp\left(\frac{Q}{T_{p}\left[t^{\prime \prime }\right]}\right)}\right){}^{\beta }\right)\frac{dT_{p}}{dt^{\prime }}\;dt^{\prime }$$

The stress in the patch is thus finally written:

(26) $$\sigma_{p}=\frac{E_{p}}{1-\nu_{p}}\left(\psi_{0}\left(\epsilon_{f,s}-\epsilon_{f,p}\right)+\left(1-\psi_{0}\right)\left(\alpha_{s}\Delta_{s}-\alpha_{p}\Delta_{p}\right)\right)$$

This equation can be used to calculate the stresses induced by a thick elastic substrate in a thin viscoelastic layer on the central region of the composite, with the two materials having different inner temperatures. In turn, this makes it possible to calculate the stresses affecting the thick elastic layer (the stone), simply by using Equation (5). The risk of delamination by adhesion failure is not covered in this analysis, and even though there is no indication from field experience to suggest that it is a problem, it is a question that will be further investigated in future work. Of course, the stresses that might contribute to delamination or decohesion also relax at about the same rate as the in-plane stresses that we examine.

Among the hypotheses involved, the bond-slip effect that can occur between the sandstone substrate and the mortar patch is ignored. Indeed, the roughness of the sandstone surface makes a mechanical interlocking of the patch with the stone likely. Furthermore, before the application of the patch, the sandstone is brushed with diluted resin to enhance the adhesion; these combined effects are assumed to prevent slippage. Another assumption concern the coefficients of thermal expansion that are taken to be constant in the range of temperature considered, as well as the elastic moduli and the Poisson’s ratio of both materials. Finally, the viscoelasticity of the thin layer is described by a stretched exponential function, that incorporates the temperature dependence of the relaxation time. The limitation of this function concerns the calculation of the stresses during a long period of time, for example when the input is a temperature evolution covering several months, and for which excessively long computation time is needed. A solution to this issue is to use a sum of exponential terms instead of a stretched exponential relaxation function, which, as described in Appendix, is far more efficient.

## 3. Materials and Methods

### 3.1. Measuring the Thermal Expansion

The thermal expansion was measured on cubes of 1 cm${}^{3}$ using a Dynamic Mechanical Analyser DMA 7e from Perkin Elmer (Perkin Elmer (Schweiz), Schwerzenbach, Switzerland). The dynamic mode is not used in this experiment, which is thus equivalent to use the device as a thermo-mechanical analyser. The temperature range went from −20 to 60 °C with a heating rate of 0.5 ${}^{\circ }C\cdot \min^{-1}$ under nitrogen flow. The force exerted by the displacement sensor was kept as low as possible, namely 10 mN thus 127 Pa.

### 3.2. Measuring the Tensile Strength of the Materials

The direct tensile strength tests were performed on a universal testing machine Zwick 1454 equipped with a 10 kN load cell (Zwick GmbH, Ulm, Germany) on six specimens of natural stone and five of artificial stone. The cylindrical samples had diameters of 20 mm and lengths of 50 mm in average. The natural stone was a Ostermundigen blue sandstone, widely spread in the historical buildings in Switzerland. The bedding has been chosen perpendicular to the strain.

The artificial stone was prepared with ground Ostermundigen blue mixed with 20% m of Plextol D512 (Synthomer Deutschland GmbH, Marl, Germany). Since the glass transition temperature of the polymer is around 20 °C, and consequently the relaxation of the stresses at room temperature is very fast, the samples were conditioned in a climatic chamber and wrapped in rockwool before the test to ensure an inner temperature lower than 15 °C, as measured by a thermocouple embedded in a sample. The strain rate adopted was 0.5 mm/min.

The tensile strength is the force at break divided by the cross-sectional area of the sample, in accordance with reference [19].

### 3.3. Measuring the Elastic Modulus by Ultrasound Pulse Velocity

The instantaneous elastic modulus $E_{0}$ represents the purely elastic mechanical property of the repair material. An approximative quantification is obtained by measuring the ultrasonic pulse velocity through a cubic sample with 5 cm edge using a Pundit Lab (Proceq SA, Schwerzenbach, Switzerland), equipped with transducers operating at a frequency of 54 kHz. The velocity is linked to the elastic modulus through the relation:

(27) $$E_{0}=\frac{\rho \times v^{2}}{K}$$

with *ρ* the density and *v* the velocity of the wave [20]; *K* is determined from the dynamic Poisson’s ratio as following:

(28) $$K=\frac{1-\nu }{\left(1+\nu )(1-2\nu \right)}$$

We take ν=0.4, in accordance with reference [21].

The evolution of the elastic modulus with the temperature has been studied between 0 and 30 °C, a range of temperature that encompasses the glass transition temperature of the acrylic polymer.

### 3.4. Measuring the Stress Relaxation

The viscoelasticity of the artificial stone is studied through the measurement of the relaxation of a load at a constant strain.

**Figure 3:**
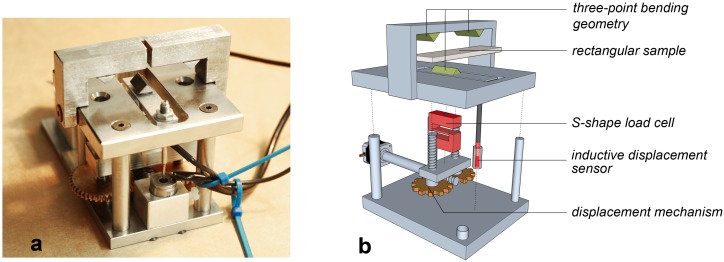
(**a**) Stress relaxation setup; (**b**) and its detailed diagram.

The relaxation tests are three-point bending tests performed with a home-made device consisting of a mechanical-loading stage and a computer-assisted control unit. The device is shown in Figure 3, and a thorough description is given by Wei [22]. The mechanical-loading stage is composed of a PRECIstep two-phase stepper motor mounted with a zero-backlash spur gearhead (1/900 ratio) (Faulhaber Precistep SA, La Chaux de Fonds, Switzerland), a Transmetra KD24S 100 N load cell (accuracy of ± 0.1%, Transmetra GmbH, Flurlingen, Switzerland), a HBM linear-variable displacement transducer W1EL/0 (accuracy of ± 2 μm, Hottinger Baldwin Messtechnik AG, Volketswil, Switzerland) and a high-precision micro-transmission system used to integrate these components. A program coded in Labview controls the rotation of the motor, displays and saves the force and displacement data.

The specimens are in the form of rectangular beams with thickness of 4 mm, width of 12 mm and length of 50 mm, while the length of the support span is 48 mm, allowing an aspect ratio of 1:3:12. The strain rate adopted was 0.045 mm/min.

**Figure 4:**
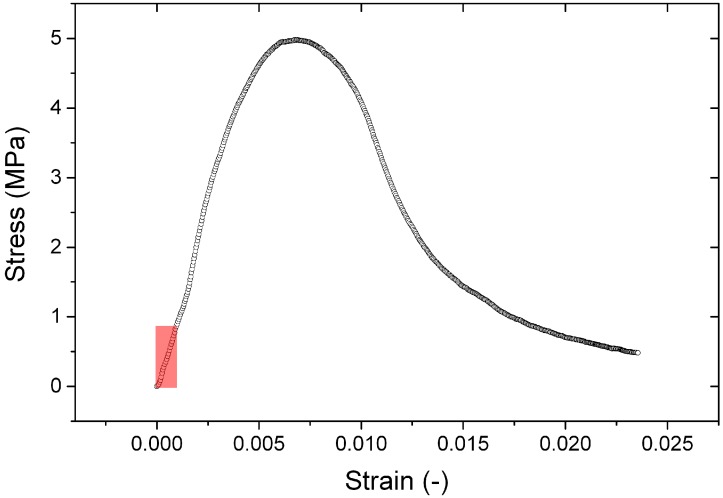
Typical stress-strain curve of the artificial stone. Note the high critical deformation. The red rectangle indicates the area of stress and strain investigated during the relaxation tests.

The stress relaxation is studied by loading the sample far from its load at break as illustrated in Figure 4.

The dependence of the relaxation on temperature is studied by repeating the measurement in a climatic chamber at temperatures from −5 to 25 °C. Higher temperatures, even though often reached during summer at the surface of the building, are not relevant because they are higher than the glass transition temperature of the polymer ($T_{g}$≈ 20 °C), where the relaxation is quasi-instantaneous.

The relative humidity in the climatic chamber has been kept below 65 %, since it has been noted that the relaxation time is strongly affected by the relative humidity; measuring the relaxation below 65 % RH ensures the relaxation to be the slowest, and thus put us in the worst case.

The Table 1 summarizes the temperatures investigated and the number of runs that have been performed for each of them. All the graphs are included as Supplementary Materials (Figure S1) to this article.

**Table 1:** Temperatures investigated and number of runs performed.

Temperatures Investigated (°C)	−5	−2.5	0	5	10	15	17.5	19	20	25
Number of Runs	1	1	2	3	2	2	2	1	2	1

### 3.5. Measuring the Temperature On-Site

**Figure 5:**
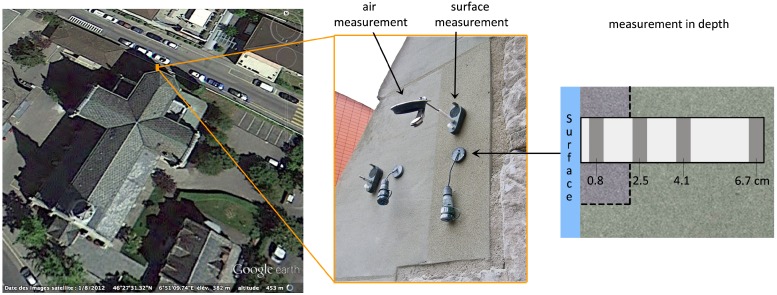
The church where the sensors have been applied in Vevey, and a close view of the equipment.

On the monument considered in this study (Catholic Church of Notre Dame de Vevey, Vevey, Switzerland), the temperature was recorded at different depths in a stone that had been repaired with the acrylic-based mortar, from July 2013 to July 2014. It was measured by capacitive Hygrochron loggers (Maxim Integrated, San Jose, CA, USA), put together in a cylindrical shape and separated by expanded Polyvinyl Chloride, a material with a low thermal conductivity (0.06 W/m.K, against 2.30 W/m.K for the molasse). The cylinder is protected by a shrink-fit tube and placed in a hole drilled in the artificial stone and the natural stone it covers. A view of the church and of the equipment installed on site is given in Figure 5. More information about the setup can be found in references [23,24].

The sensors function through the 1-Wire protocol, which permits as many wires as sensors plus one for the ground, hence keeping as low as possible the physical impact of any sensor on the measurement, as the quality could be reduced by water infiltration or thermal heating of the wires. Moreover, each sensor being autonomous, the need for a central acquisition system is avoided, thus reducing the invasive nature of the installation. The sensors measure the temperature in a range of −20 to 85 °C with a resolution of 0.06 °C and an accuracy of 0.5 °C between −10 and 65 °C, that extends beyond the conditions observed during the campaign. The sampling interval was 30 min.

The best location to position the sensors was determined by its potential of receving sun on a repair. This was identified on the buttress of an apse, approximately six meters above the ground. The selected location is facing west and receives the warm sun of the afternoon during summer, causing large temperature variations interesting for our purpose.

## 4. Results

### 4.1. Thermal Expansion

**Figure 6:**
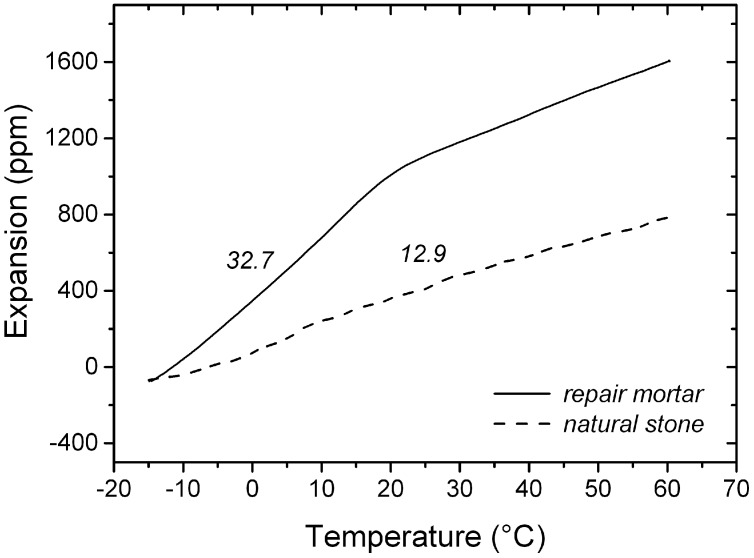
Thermal expansion of the natural stone (dash line) and the artificial stone (full line) measured by DMA. The coefficient of thermal expansion is reported.

The thermal expansion coefficient of the artificial stone, before the glass transition temperature $T_{g}$, is 2.5 times higher than that of the natural stone. Above this temperature (around 20 °C), the measurement shows a decrease of the thermal expansion coefficient, which is attributed to relaxation in the dilatometer and is thought not to reflect the real physical volume expansion that accompanies the glass transition of amorphous materials [15]. The measurement is shown in Figure 6 and the values reported in Table 2.

**Table 2:** Coefficients of thermal expansion of the artificial stone and the natural stone, measured by thermo-mechanical analysis in a DMA.

Material	Coefficient of Thermal Expansion (10^−6^·K^−1^)
Artificial Stone	32.7
Natural Stone	12.9

### 4.2. Tensile Strength

The tensile strength of the repair material and the natural stone (Ostermundigen Blue) is reported in the Table 3. When wet, the sandstone softens dramatically and loses 2/3 of its strength in the dry state.

**Table 3:** Tensile strength of the repair material and of the Ostermundigen Blue.

Material	Tensile Strength (MPa)	
Artificial Stone	2.7	
Natural Stone (Ostermundigen Blue Sandstone), Bedding ⊥ to Load	Dry	1.0
	Wet	0.3

### 4.3. Evolution of the Elastic Modulus with the Temperature

**Figure 7:**
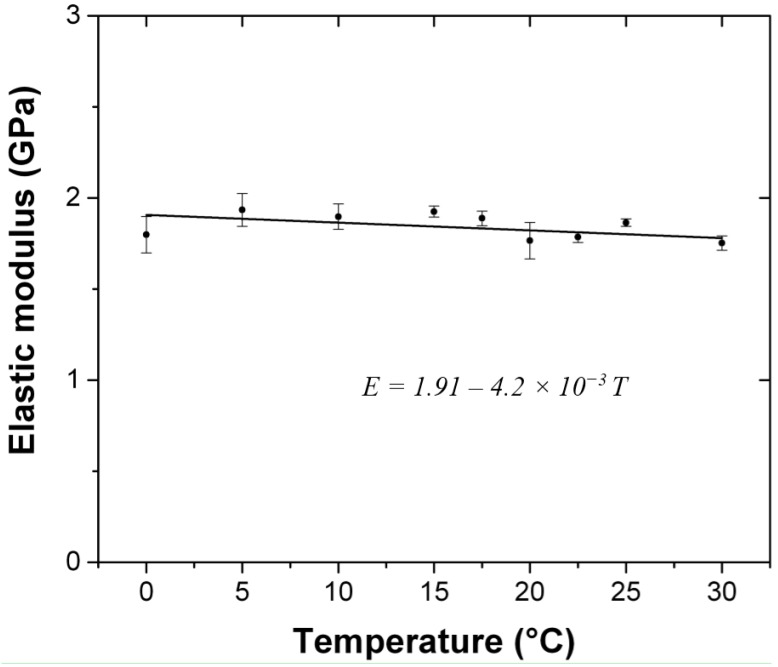
Evolution of the instantaneous elastic modulus with the temperature, calculated from the measurement of ultrasonic pulse velocity.

The elastic modulus of the artificial stone, as calculated from the measurement of ultrasound pulse velocity, is not affected by the $T_{g}$ (20 °C), as shown in Figure 7. A regression line shows a decrease with the temperature in the proportion of 4.2 MPa/°C, in the range of 0 to 30 °C. However, the uncertainty is large and we need to assess whether this trend is significant or not. To answer this question we used a statistical test, of which the theoretical fundations can be found in reference [25] and which has already been successfully used in reference [26]. The complete calculation can be found in the Supplementary Materials. As a result we find that this confidence level is low (*p* = 69%) so that it is not possible to answer “no” with confidence to the statement “does temperature have no effect on the elastic modulus?”. That implies that there may be a temperature effect, but that if it is the case, it is too low to be detected considering the experimental error of our measurements. For this reason we consider that the elastic modulus is independent of temperature and treat it as a constant in our subsequent calculations.

### 4.4. Viscoelasticity

#### 4.4.1. Stress Relaxation Function

The relaxation function we used to describe the decay of the stress in the relaxation experiment is a stretched exponential function:

(29) $$\psi \left[t\right]=\psi_{0}+\left(1-\psi_{0}\right)\exp\left(-{\left(t/\tau \right)}^{\beta }\right)$$

where *ψ* is the dimensionless relaxation function; $\psi_{0}$ the residual fraction of the stress that does not relax; *τ* the relaxation time; and *β* an exponent (0<β<1). The relaxation modulus is the product of the instantaneous elastic modulus $E_{0}$ with the relaxation function:

(30) $$E\left[t\right]=E_{0}\left(\psi_{0}+\left(1-\psi_{0}\right)\exp\left(-{\left(t/\tau \right)}^{\beta }\right)\right)$$

The temperature dependence is reflected in the relaxation time *τ*; while $E_{0}$, $\psi_{0}$ and *β* are kept constant in the temperature range considered. This approach is based on a general description of relaxation processes for glassy systems such as glasses, spin-glasses, polymers, viscous fluids and disordered dielectrics [27].

**Figure 8:**
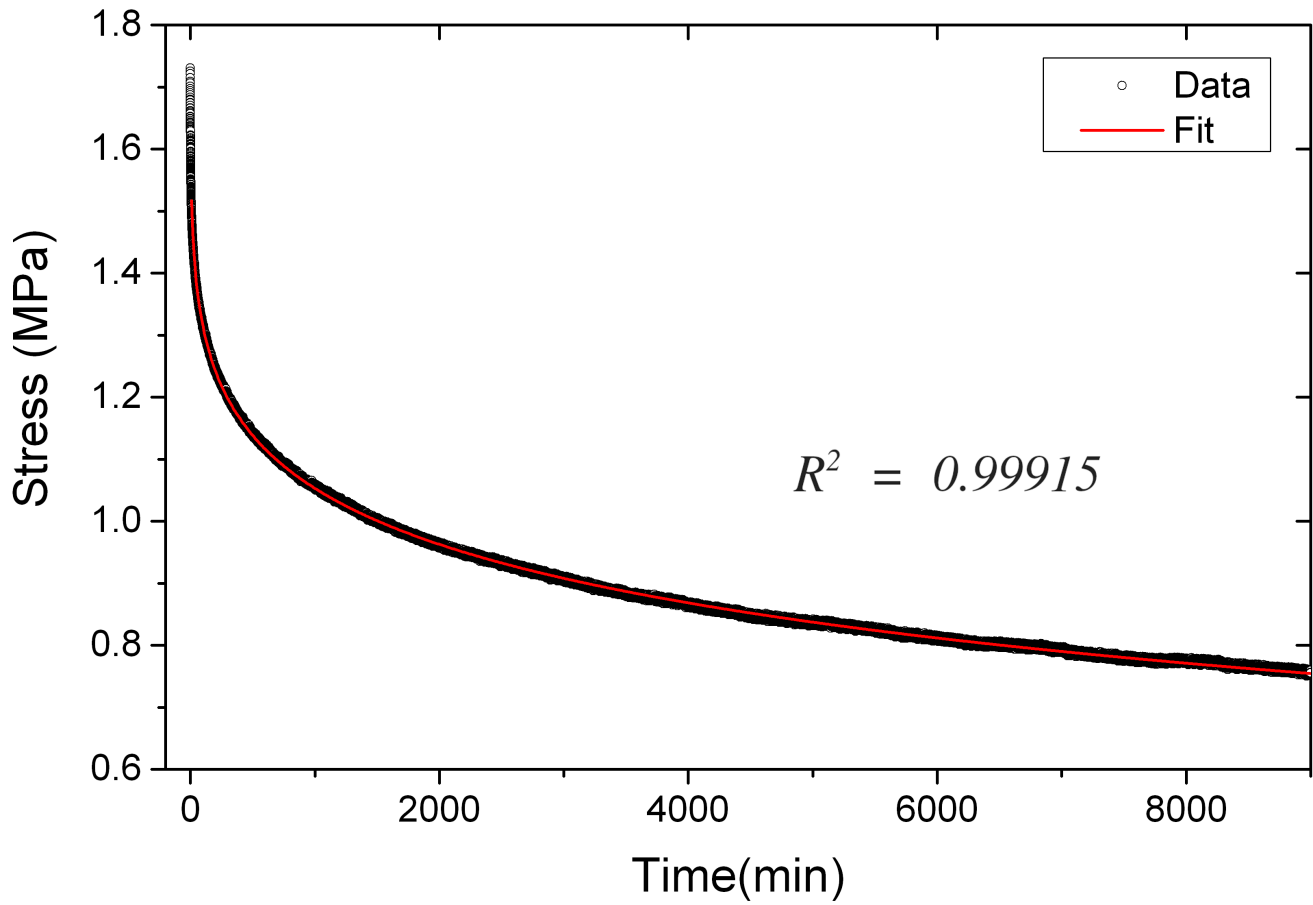
Experimentally-determined stress relaxation curve (made at −5 °C) and its fit with a stretched exponential relaxation function.

An example of a fit of a stress relaxation curve with the use of Equation (30), is given in Figure 8. It is visible here that the stretched exponential function captures better the long time relaxation processes than the short times, which are also subject to more experimental inaccuracy.

#### 4.4.2. Parameters from the Fit of the Stress Relaxation Curves

The determination of an adequate relaxation function enables the complete analysis of the stress relaxation experiment, which can be found in Appendix. The evolution of the stress during the test, when the strain is kept constant, can be described by the following equation:

(31) $$\sigma_{z}\left[t\right]=E_{0}{\dot{\epsilon }}_{z0}\tau \frac{\psi_{0}t_{\mathrm{end}}}{\tau }+\left(\frac{1-\psi_{0}}{\beta }\right)\left(\Gamma \left[\frac{1}{\beta },\left(\frac{t-t_{\mathrm{end}}}{\tau }\right){}^{\beta }\right]-\;\Gamma \left[\frac{1}{\beta },{\left(\frac{t}{\tau }\right)}^{\beta }\right]\right)\;$$

where the parameters ${\dot{\epsilon }}_{z0}$ the constant strain rate applied at the beginning of the test; and $t_{\mathrm{end}}$, the time at which the strain becomes constant, are known. The fit of the data, performed through the NonLinearModelFit algorithm of *Mathematica* 10.2 (Wolfram Research, Champaign, IL, USA), provides thus $E_{0}$, the elastic modulus of the artificial stone, *τ*, the relaxation time, *β*, the exponent of the stretched exponential relaxation function, and $\psi_{0}$ the residual fraction of the stress that does not relax.

The relaxation time *τ* shows an Arrhenius-type dependence with an activation energy of 327.2 kJ/mol.

**Figure 9:**
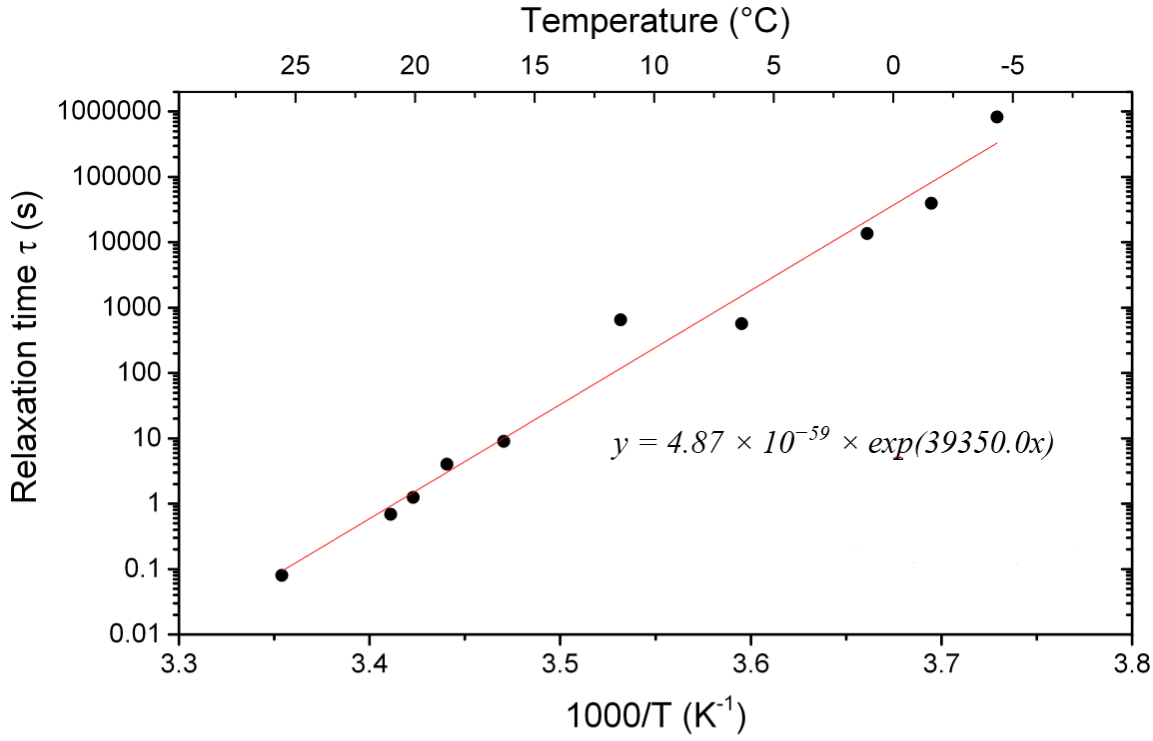
Arrhenius-type dependence of the relaxation time *τ*.

The parameters found from the fits are summarized in Table 4.

**Table 4:** Parameters describing the viscoelasticity of the artificial stone and its dependence on temperature, as obtained from the fit of the relaxation curves.

$E_{0}$ (GPa)	*β* (-)	*τ*(s)	$\psi_{0}$ (-)
2.03	0.206	$4.87\times 10^{-59}\exp\left({}^{3.94\times 10^{4}}\;/\;T\right)$	$6.95\times 10^{-3}$

### 4.5. Temperature Profile, Temperature Amplitude and Heating/Cooling Rate

**Figure 10:**
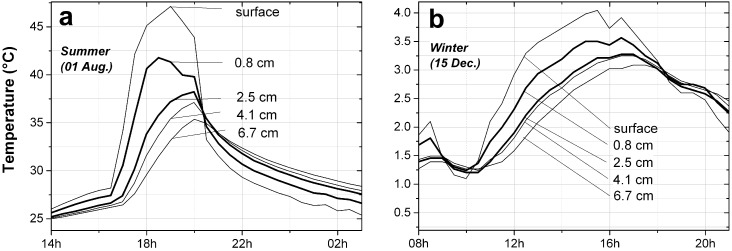
Temperature gradient in the depth of the wall. In bold, the temperatures used in the calculation. (**a**) In summer, a difference of 13.8 °C has been measured between the surface and the first 7 cm of the wall; (**b**) In winter, the gradient is much smaller, on the order of 1.5 °C in the first 7 cm of the wall.

**Figure 11:**
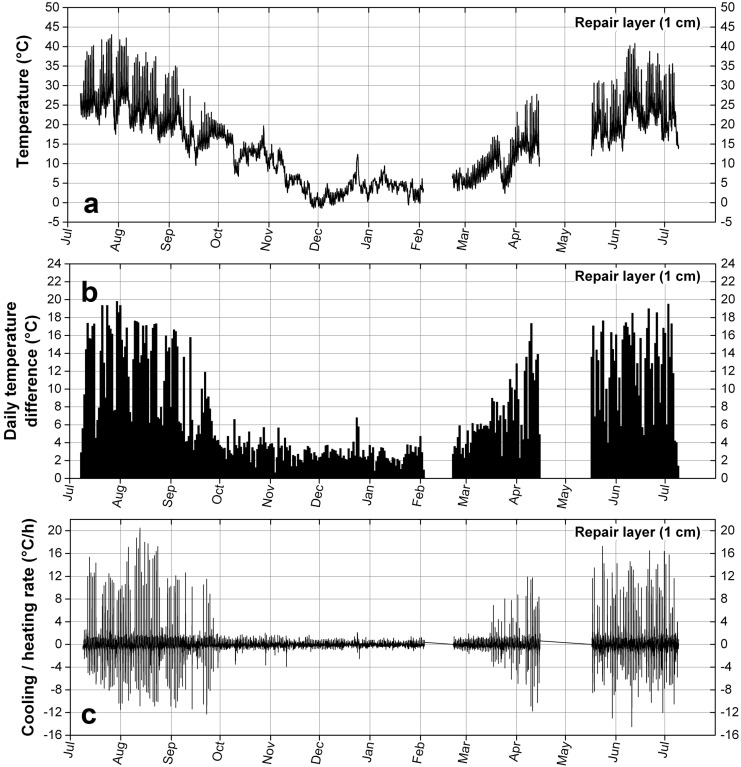
Temperature data measured in Notre-Dame de Vevey in the repair layer at a depth of 1 cm. (**a**) Temperature profile; (**b**) Temperature amplitude (daily temperature difference); (**c**) Rate of temperature variation.

**Figure 12:**
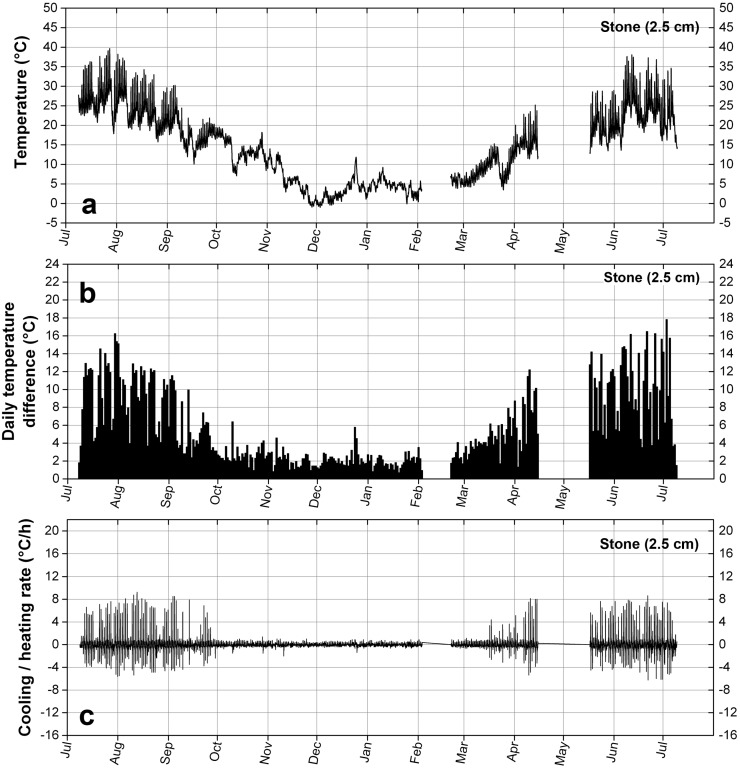
Temperature data measured in Notre-Dame de Vevey in the stone, below the interface with the repair layer, at a depth of 2.5 cm. (**a**) Temperature profile; (**b**) Temperature amplitude (daily temperature difference); (**c**) Rate of temperature variation.

The development of thermal stresses depends on the magnitude of the temperature variations and, due to the time-dependant mechanical properties of the mortar, on how fast these variations occur. Since the stone is not directly exposed to the outside but is covered by the repair layer, it exists a gradient of temperature in the depth of the wall that affects both the amplitude of the temperature variations and the rate at which they develop in the two materials. This temperature difference can reach 13.8 °C in summer between the surface of the repair layer and the first seven centimeters of the wall, as shown in Figure 10a. If we consider that the temperature representative of the repair layer is the volume-averaged temperature, namely the one measured at 0.8 cm in the middle of the patch, and the temperature of the stone the temperature at a depth just below the patch, at 2.5 cm, we find that the stone can be 6 °C colder than the repair mortar during a hot summer day. In winter this difference reduces to 0.5 °C. Nevertheless, it justifies to consider two different temperatures in the computation of the thermal stresses. For these reasons the temperature profile, the daily temperature amplitude and the heating and cooling rate are reported separately for each material, in Figure 11 for the repair layer and in Figure 12 for the stone.

The measurement of the temperature in the two materials, in Figure 11a and Figure 12a, from July 2013 to July 2014, clearly shows an underlying seasonal variation. The repair layer experiences high temperatures in summer up to 43 °C and low temperatures in winter down to −1.5 °C, while the temperature of the stone, below the repair layer, goes from 39.7 in summer to −1 °C in winter.

The most frequent periodical variations of temperature are the ones occurring daily, namely the difference between the maximum and minimum temperatures due to the alternation of night and day. They are displayed in Figure 11b and Figure 12b. To July and August are associated daily temperature differences that can reach 19.8 °C in the repair material, varying around a mean temperature of 24.2 °C, a temperature associated with fast relaxation of stresses because higher than its $T_{g}$. The temperature difference in summer can reach 17.8 °C in the stone. On the other hand in winter, or more exactly in a period of six months extending from October to March, the maximum daily temperature differences are much smaller, not larger than 6.7 °C, and they are similar in the two materials.

These daily variations of temperature give rise to the highest heating and cooling rates, as shown in Figure 11c and Figure 12c. In the repair layer the temperature can rise at a rate of 20.5 °C/h and drop at a rate of −14.5 °C/h, while the highest heating rate in the stone is 9.3 °C/h and the highest cooling rate is −6.2 °C/h. High heating and cooling rates can be associated to a period extending from March to October, while from October to March the rates are much lower. It can also be noted that the heating rates are higher than the cooling rates: during the warmer period the stone is able to warm up fast, due to the direct sunlight radative heating, while the cooling down is achieved through slower processes of convection and conduction. In the colder period they are nearly similar due to the absence of sun.

## 5. Calculation of the Thermal Stresses

The computation of the thermal stresses is achieved by combining the material properties measured in the laboratory with the measurements of the thermal conditions on-site, presented in Section 4, through the analytical description of the stress in the repair layer presented in Equation (26). The code used for the calculation is given as a supplementary material to this article. The thermal stresses in a viscoelastic material depend on the history of heating and cooling events that determines its current state of stress and strain. For this reason, the stresses are calculated starting from a temperature where the stresses and strains are the lowest possible, which is found at a temperature above the $T_{g}$ (20 °C) of the acrylic mortar, a situation common during summer.

### 5.1. In the Repair Mortar

**Figure 13:**
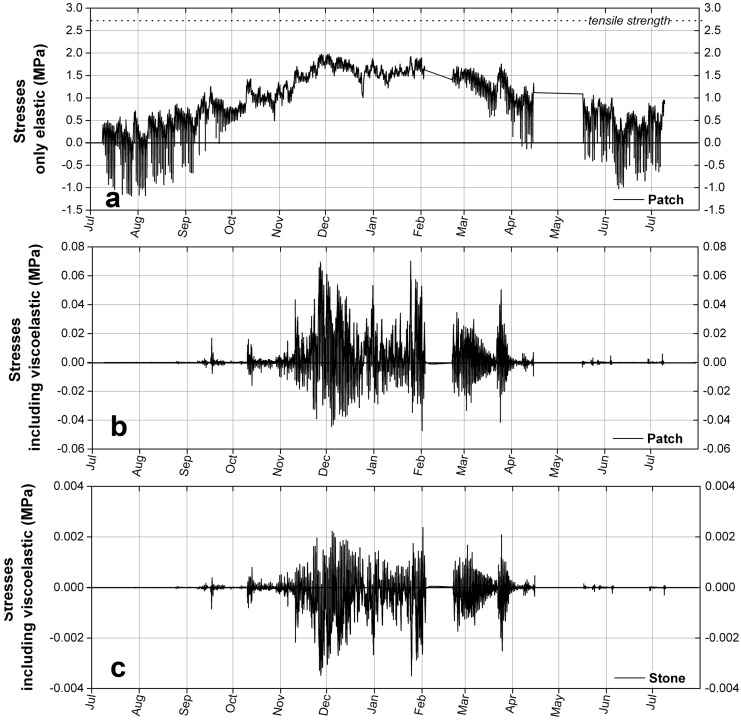
Stresses calculated from the temperature data measured in Vevey. (**a**) Stresses, without including the viscoelasticity, in the repair mortar, compared to its tensile strength; (**b**) Stresses, including the viscoelasticity, in the repair mortar; (**c**) Induced stresses in the stone.

The thermal stresses in the repair mortar are presented in Figure 13a,b. A comparison between the stresses that do not consider the viscoelasticity of the repair material and the stresses that do include it is very useful to grasp the importance of this relaxation phenomenon and the magnitude of the stresses it relaxes.

If the repair material were considered to be purely elastic, the thermal stresses in the repair layer would be calculated with the Equation (11). This analysis, of which the results are presented in Figure 13a shows that the stresses are mostly tensile (a positive stress means tensile stress while a negative one means compressive) and would reach 2.0 MPa, thus 74% of the tensile strength of the repair material, which would place the mortar in a situation of fracture through repetition of the stresses throughout the years. However, taking into consideration the viscoelastic behaviour of the acrylic-based mortar reveals a totally different story, which is illustrated in Figure 13b. The maximum viscoelastic stresses in the repair material is then only 1/38 of the maximum elastic stresses, far from the tensile strength of the repair material. It is clear that the viscoelasticity of the acrylic-based mortar, that results from the relatively low $T_{g}$ of the acrylic resin, associated with the slow cooling rates observed on-site, is a very effective mechanism that works against the development of high stresses and for the mecanical compatibility of the two materials.

A closer look at the viscoelastic stresses in the repair mortar shows that the stresses are not only tensile. The relaxation of the stresses after a decrease of the temperature leads to the formation of compressive forces during a subsequent heating. The forces are then alternatively tensile and compressive, and of very low magnitude. Moreover, it can be seen that no stresses are built up during the warm summer period, despite the high temperature amplitude and the high cooling rates. This is a result of the fast relaxation of stresses at these temperatures. In contrast, the thermal stresses are increasing during the colder months, when the mortar layer shrinks more than the stone substrate, inducing tensile forces in the repair layer. Theses stresses are less relaxed at these temperatures, but still remain far lower than the strength of the material.

### 5.2. In the Natural Stone

The stresses in the natural stone are a fraction of the stresses in the repair layer, due to their very different thicknesses, according to Equation (5). This equation states that the sign of the stresses in the stone is the opposite of the stress in the repair layer: if tensile stresses develop in the repair layer, compressive forces arise in the stone and vice versa. The results of the calculation are presented in Figure 13c. The maximum tensile stress induced in the stone is 0.0015 MPa, which represents only 0.5% of its tensile strength in the wet state, thus in a state where the stone is considerably less resistant than in the dry state. The maximum compressive stress in the stone is 0.0027 MPa, far from its compressive strength. These values of stresses are extremely safe for the stone. However, providing that the winter 2013–2014 is not considered a cold winter, and that the highest stresses are created in winter, the values of stresses calculated above may not be representative of what could be the worst situation for the materials. A worst case scenario is addressed in the next section.

### 5.3. Thermal Stresses in the Worst Conditions

#### 5.3.1. Case of La Brévine

**Figure 14:**
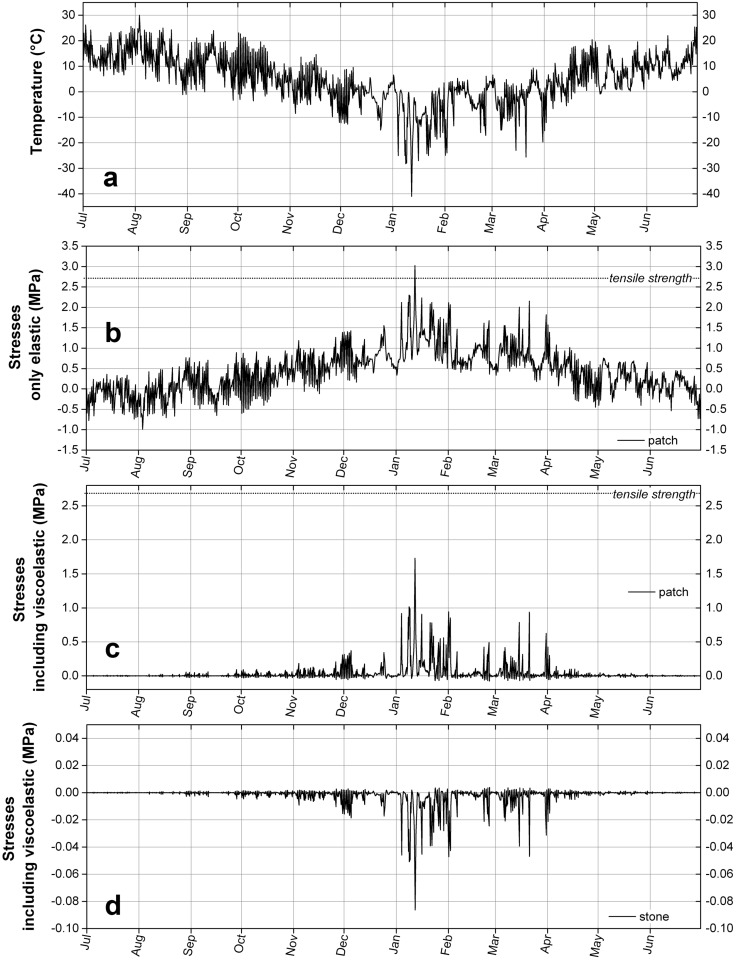
(**a**) Temperature in La Brévine in January 1987; (**b**) Calculation of the thermal stresses in the repair material, without considering its viscoelasticity; (**c**) Calculation of the expected viscoelastic stresses in the repair material; (**d**) Calculation of the thermal stresses in the natural stone.

In Switzerland, the coldest winter ever recorded happened in 1987 in a village called La Brévine, also known as “the Swiss Siberia”, in the canton of Neuchâtel. Between 10 and 12 January, the temperature of the air 2 m from the ground dropped from 0 to −41 °C, with a maximum cooling rate of −1.8 °C/h, as seen in Figure 14a. Since in winter the radiative heating of the sun does not warm up the surface, the temperature of the repair layer (at least at the surface) is close to that of the air, which makes the record of temperature eligible to serve as the temperature, though exaggerated, of the two materials. Under such conditions, a repair done with the acrylic-based mortar would have experienced a tensile stress of 1.7 MPa while the stone would have endured a compressive stress of 0.09 MPa, as shown in Figure 14b,c. Although such stresses cannot harm the stone, they do on the other hand amount to 63% of the tensile strength of the mortar. At these temperatures and cooling rates, the relaxation of the stresses is slower and the material approaches a purely elastic behaviour; however, if we consider the whole history of the stresses from the previous summer until this cold event, the viscoelasticity of the mortar nevertheless prevents tensile stresses from reaching values in excess of the tensile strength. Therefore, even for these exceptional conditions, we see here that a sudden decrease of temperature with such a large temperature difference would not result in fracture neither in the natural stone nor in the artificial stone. The case of thermal fatigue, that relies on repeated heating-cooling cycles and to a subsequent gradual weakening of the stone, is highly unlikely since these extreme cold events have nowadays a repetition probability lower than the expected lifetime of a repair [28], which is, according to Torney, of 30 years [2].

#### 5.3.2. Hypothetical Conditions Leading to Fracture

**Figure 15:**
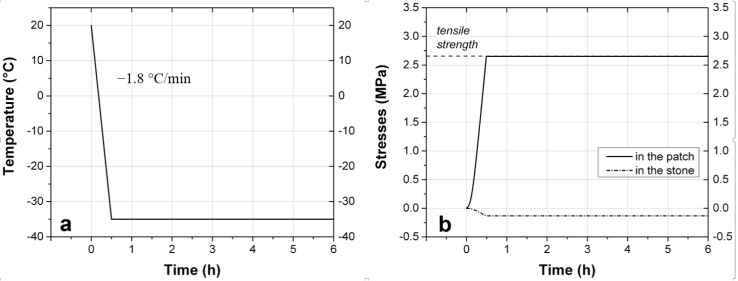
(**a**) The decrease of temperature that could lead to damage in the mortar; (**b**) The stresses induced in the stone and in the mortar by such a decrease in temperature, compared to the tensile strength of the mortar.

Thermal stresses reaching the tensile strength of the repair mortar could however be obtained by a sudden drop of temperature from 20 to −35 °C happening in 30 min, thus at a rate of −1.8 °C/min. This situation is described by the graphs shown in Figure 15. Even though such a cooling rate has been measured at the surface of rocks in nature [29], it has only been observed for a decrease of temperatures of a few degrees, and never on an amplitude of 55 °C. Furthermore, it can not happen in all likelihood at a depth of few centimeters in a wall. However, this example *reductio ad absurdum* has the virtue of showing that even though the thermal stresses could theoretically exceed the tensile strength of the mortar, the induced compressive stresses in the natural stone are still below the compressive strength of the natural stone.

## 6. Conclusions

We have examined the question of thermo-mechanical compatibility of an acrylic-based repair mortar and a sandstone, in case of a typical reprofiling, that is a thick stone substrate which imposes its deformation on a thin layer of repair material. The repair mortar is characterized by a low strength and elastic modulus, as well as a high viscoelastic relaxation. Such properties make it adequate for patches, but the creep characteristics associated with viscoelasticity do not make it suitable for any structural application. Given the large thermal expansion coefficient mismatch between the acrylic-based mortar and the sandstone it aims to repair, the question of the magnitude of the thermal stresses that could be induced in the two materials is legitimate. Indeed, a calculation that would treat the repair mortar as a purely elastic material would deliver a result of stress higher than the tensile strength of the repair material. However, since the acrylic-based mortar displays viscoelastic properties associated with a relatively low $T_{g}$ (20 °C), the relaxation processes specific to this type of material have to be considered. The amplitude of the temperature variations, the value of the coldest temperature reached and the rate at which the temperature variations occur all play a role in the magnitude of the thermal stresses. Even by considering the worst case-scenario, the analysis shows that the thermal stresses are not high enough to harm the repair material, and more importantly, are insignificant for the integrity of the historical substrate. The key role of the glass transition temperature has to be stressed: choosing an acrylic polymer with a $T_{g}$ in the range of temperatures found on-site allows to benefit from an interesting property of the polymer from a conservation point of view, namely its reversibility, without having to suffer from one of its drawback, a high thermal expansion coefficient.

With respect to the ultimate durability of such patches other issues also have to be considered, for example water migration and salt crystallization might be modified around the hydrophobic patch.

## Figures and Tables

**Figure A1:**
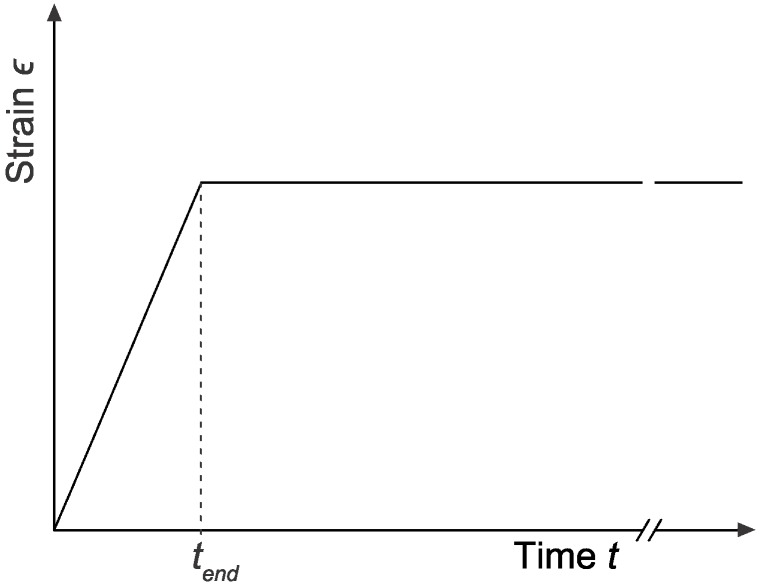
Strain applied during the stress relaxation experiment.

## Supplementary Materials

The following are available online at www.mdpi.com/1996-1944/9/1/56/s1.

## Appendix

### Fitting the Stress Relaxation Curves

This appendix presents the theory for the analysis of the stress relaxation measurements. It uses the relaxation theory for viscoelastic materials of which a comprehensive presentation can be found in reference [15] and on which this work is based.

The fit of the data from the stress relaxation experiment depends on the instantaneous elastic modulus $E_{0}$, the relaxation time, *τ*, the exponent, *β* and the residual fraction of the stress that does not relax, $\psi_{0}$.

If a constant uniaxial strain, $\epsilon_{z}$, is instantaneously applied to a viscoelastic material, the resulting stress, $\sigma_{z}$, relaxes with time, *t*, and the relaxation function, *ψ*, is defined by

(32) $$\psi \left[t\right]=\frac{\sigma_{z}\left[t\right]}{\sigma_{z}\left[0\right]}$$

In our experiments, the deflection is however not instantaneous and we must account for the finite time taken to apply it, as shown in Figure A1. For this, we consider the constant strain rate, ${\dot{\epsilon }}_{z0}$, that is applied from *t* = 0 to *t* = $t_{\mathrm{end}}$:

(33) $${\dot{\epsilon }}_{z}\left[t\right]={\dot{\epsilon }}_{z0}H\left[t_{\mathrm{end}}-t\right]$$

where *H* is the Heaviside (unit step) function,

(34) $$H\left[t_{\mathrm{end}}-t\right]=\begin{array}{cc} \{ & \begin{array}{cc} 1 & ,0\leq t\leq t_{\mathrm{end}}\\ 0 & ,t>t_{\mathrm{end}} \end{array} \end{array}$$

So that the strain rate is constant until the final deflection is reached, at $t_{\mathrm{end}}$, and zero afterwards.

The axial stress in the beam, $\sigma_{z}$, is given by the following hereditary integral [16,30,31]:

(35) $$\sigma_{z}\left[t\right]=E_{0}\int_{0}^{t}\psi \left[t-t^{\prime }\right]{\dot{\epsilon }}_{z}\left[t^{\prime }\right]dt^{\prime }$$

where $E_{0}$ is the instantaneous (infinite frequency) modulus; and *ψ* is the stress relaxation function. This equation describes the stress at any time *t* during the relaxation test, by considering any incremental deflection from its application time *t’* until the time *t* considered. For any time *t’* > *$t_{\mathrm{end}},$* at which no incremental deflection is applied, ${\dot{\epsilon }}_{z}$ is equal to zero. This equation thus addresses the non-instantaneous deflection issue by quantifying the relaxation that takes place during the time of application of the deflection.

By imposing a deflection with a constant strain rate until *t* = $t_{\mathrm{end}}$, the final strain is:

(36) $$\epsilon_{z0}={\dot{\epsilon }}_{z0}t_{\mathrm{end}}$$

The relaxation function is approximated by a stretched exponential:

(37) $$\psi \left[t\right]=\psi_{0}+\left(1-\psi_{0}\right)\exp\left(-{\left(t/\tau \right)}^{\beta }\right)$$

where *β* is a constant that lies between 0 and 1; and $\psi_{0}$ is the residual fraction of the stress that does not relax.

The use of a stretched exponential is advantageous due to its satisfactory approximation of the relaxation with just three adjustable parameters $\psi_{0}$, *τ* and *β*. On the other hand, the relaxation could be precisely fitted with a sum of exponentials:

(38) $$\psi \left[t\right]=\sum_{j=1}^{N}w_{j}\exp\left(-t/\tau_{j}\right)$$

but this would require six or more relaxation times $\tau_{j}$ [15], often arbitrarily chosen, and the same number of weighing factors $w_{j}$; these latter indicating the relative importance of each relaxation time. The higher number of parameters would increase the complexity of the mathematical description. The satisfactory accuracy of the approximation with a stretched exponential function has first been recognized by Kohlrausch in 1854 for the decay of residual charge in a Leyden jar [32]. Since then it has been used to characterize relaxation processes in many different systems, like the viscoelastic behavior of glasses [15,33] , the fluorescence decay of biological tissues [34] , or even the distribution of urban agglomeration sizes or the distribution of light emission from galaxies, among the various uses of this function evidenced by Laherrère and Sornette [35].

To simplify the notation, we write:

(39) $$\theta =t/\tau ,\theta_{\mathrm{end}}=t_{\mathrm{end}}/\tau \;,\epsilon_{z0}={\dot{\epsilon }}_{z0}\theta_{\mathrm{end}}\tau$$

If the strain rate is given by Equation (33), Equation (35) becomes:

(40) $$\sigma_{z}\left[\theta \right]=E_{0}{\dot{\epsilon }}_{z0}\tau \int_{0}^{\theta }\psi \left[\theta -\theta^{\prime }\right]H\left[\theta_{\mathrm{end}}-\theta^{\prime }\right]d\theta^{\prime }=\frac{E_{0}\epsilon_{z0}}{\theta_{\mathrm{end}}}\int_{0}^{\theta }\psi \left[\theta -\theta^{\prime }\right]H\left[\theta_{\mathrm{end}}-\theta^{\prime }\right]d\theta^{\prime }$$

Two situations must be considered for the above equation, depending on whether the time at which relaxation is considered is smaller or greater than the duration of application of the deflection.

This leads to the following two expressions:

(41) $$\frac{\sigma_{z}}{E_{0}\epsilon_{z0}}=\begin{array}{cc} \{ & \begin{array}{cc} \frac{\psi_{0}\theta }{\theta_{\mathrm{end}}}+\left(\frac{1-\psi_{0}}{\beta \theta_{\mathrm{end}}}\right)\left(\Gamma \left[\frac{1}{\beta }\right]-\Gamma \left[\frac{1}{\beta },\theta^{\beta }\right]\right) & ,\theta <\theta_{\mathrm{end}}\\ \psi_{0}+\left(\frac{1-\psi_{0}}{\beta \theta_{\mathrm{end}}}\right)\left(\Gamma \left[\frac{1}{\beta },\left(\theta -\theta_{\mathrm{end}}\right){}^{\beta }\right]-\;\Gamma \left[\frac{1}{\beta },\theta^{\beta }\right]\right) & ,\theta >\theta_{\mathrm{end}} \end{array} \end{array}$$

where Γ[*a*,*z*] is the incomplete gamma function

(42) $$\Gamma \left(a,z\right)=\int_{z}^{\infty }t^{a-1}e^{-t}dt$$

and Γ[*z*] is the complete gamma function

(43) $$\Gamma \left[z\right]=\int_{0}^{\infty }t^{z-1}e^{-t}dt$$

The fit is applied to the data following the end of the down-drive, when the strain is constant:

(44) $$\sigma_{z}\left[\theta \right]\;=E_{0}{\dot{\epsilon }}_{z0}t_{\mathrm{end}}\left(\psi_{0}+\left(\frac{1-\psi_{0}}{\beta \theta_{\mathrm{end}}}\right)\left(\Gamma \left[\frac{1}{\beta },\left(\theta -\theta_{\mathrm{end}}\right){}^{\beta }\right]-\;\Gamma \left[\frac{1}{\beta },\theta^{\beta }\right]\right)\right),\theta >\theta_{\mathrm{end}}$$

Which can be written in term of time *t*:

(45) $$\sigma_{z}\left[t\right]=E_{0}{\dot{\epsilon }}_{z0}\tau \left(\frac{\psi_{0}t_{\mathrm{end}}}{\tau }+\left(\frac{1-\psi_{0}}{\beta }\right)\left(\Gamma \left[\frac{1}{\beta },\left(\frac{t-t_{\mathrm{end}}}{\tau }\right){}^{\beta }\right]-\;\Gamma \left[\frac{1}{\beta },{\left(\frac{t}{\tau }\right)}^{\beta }\right]\right))\;,t>t_{\mathrm{end}}\right.$$

The strain rate, ${\dot{\epsilon }}_{z0}$, and the time at the end of the downdrive, $t_{\mathrm{end}}$, are known, so the fitting parameters are the instantaneous modulus, $E_{0}$, relaxation time, *τ*, exponent, *β* and residual fraction of the stress that does not relax, $\psi_{0}$.

### Calculation of the Stresses Using a Sum of Exponential Relaxation Function

The long computation time required to calculate the stresses using a stretched exponential function makes it unsuitable for long-period analysis of the stress. It can be approximated by a sum of exponentials which makes the computation more efficient.

Let the stress relaxation function be represented by a sum of exponential terms,

(46) $$\exp\left(-{\left(t/\tau \right)}^{\beta }\right)\approx \sum_{j=1}^{N}w_{j}\exp\left(-t/\tau_{j}\right)$$

in which $\tau_{j}$ are discrete relaxation times; and $w_{j}$ are weighting factors found by specifying *N* values of *t*. The higher *N* is, or the more relaxation times $\tau_{j}$ are used, the better is the description of the relaxation curve. For our purpose, a good agreement can be found between the two relaxation curves with *N* = 20.

If the system exhibits thermorheological simplicity, then each of the relaxation times has the same temperature dependence:

(47) $$\tau_{j}\left[t\right]=\tau_{0j}\exp\left(Q/T_{p}\left[t\right]\right)$$

and the integrals in the stress equation (Section 2, Equation (24) and Equation (25)) become:

(48) $$\begin{array}{cc} \Delta_{m}\left[t\right] & \equiv \int_{0}^{t}\exp\left(-\left(\int_{t^{\prime }}^{t}\frac{dt^{{}^{\prime \prime }}}{\tau [t^{\prime \prime }]}\right){}^{\beta }\right)\frac{dT_{m}}{dt^{\prime }}dt^{\prime }\;\\ & \approx \;\sum_{j=1}^{N}w_{j}\int_{0}^{t}\exp\left(-\int_{t^{\prime }}^{t}\frac{dt^{\prime \prime }}{\tau_{j}\left[t^{\prime \prime }\right]}\right)\frac{dT_{m}}{dt^{\prime }}dt^{\prime }\;,m=s,p \end{array}$$

If we define

(49) $$\Delta_{m,j}\left[t\right]=\int_{0}^{t}\exp\left(-\int_{t^{\prime }}^{t}\frac{dt^{\prime \prime }}{\tau_{j}\left[t^{\prime \prime }\right]}\right)\frac{dT_{m}}{dt^{\prime }}dt^{\prime }\;,m=s,p$$

then

(50) $$\Delta_{m}\left[t\right]=\;\sum_{j=1}^{N}w_{j}\Delta_{m,j}\left[t\right]\;,m=s,p$$

and the relaxational part of the differential strain of Equation (26) is

(51) $$\Delta \epsilon_{f,r}\left(t\right)\equiv \alpha_{s}\Delta_{s}-\alpha_{p}\Delta_{p}=\alpha_{s}\sum_{j=1}^{N}w_{j}\Delta_{s,j}\left[T_{s}\left[t\right]\right]-\alpha_{p}\sum_{j=1}^{N}w_{j}\Delta_{p,j}\left[T_{p}\left[t\right]\right]$$

To find the stress, we only need to evaluate 2N equations of the form of Equation (49), using the procedure described by Scherer in reference [36]. For the numerical evaluation, we want to find the strain at time t+Δt from the strain at *t*, so we expand the integrals as follows:

(52) $$\begin{array}{cc} \Delta_{m,j}\left[t+\Delta t\right] & =\int_{0}^{t+\Delta t}\exp\left(-\int_{t^{\prime }}^{t+\Delta t}\frac{dt^{\prime \prime }}{\tau_{j}\left[t^{\prime \prime }\right]}\right)\frac{dT_{m}}{dt^{\prime }}dt^{\prime }\;,m=s,p\\ & =\int_{t}^{t+\Delta t}\exp\left(-\int_{t^{\prime }}^{t+\Delta t}\frac{dt^{\prime \prime }}{\tau_{j}\left[t^{\prime \prime }\right]}\right)\frac{dT_{m}}{dt^{\prime }}dt^{\prime }+\int_{0}^{t}\exp\left(-\int_{t^{\prime }}^{t+\Delta t}\frac{dt^{\prime \prime }}{\tau_{j}\left[t^{\prime \prime }\right]}\right)\frac{dT_{m}}{dt^{\prime }}dt^{\prime }\\ & =\int_{t}^{t+\Delta t}\exp\left(-\int_{t}^{t+\Delta t}\frac{dt^{\prime \prime }}{\tau_{j}\left[t^{\prime \prime }\right]}\right)\exp\left(-\int_{t^{\prime }}^{t}\frac{dt^{\prime \prime }}{\tau_{j}\left[t^{\prime \prime }\right]}\right)\frac{dT_{m}}{dt^{\prime }}dt^{\prime }\\ & +\int_{0}^{t}\exp\left(-\int_{t}^{t+\Delta t}\frac{dt^{\prime \prime }}{\tau_{j}\left[t^{\prime \prime }\right]}\right)\exp\left(-\int_{t^{\prime }}^{t}\frac{dt^{\prime \prime }}{\tau_{j}\left[t^{\prime \prime }\right]}\right)\frac{dT_{m}}{dt^{\prime }}dt^{\prime } \end{array}$$

The inner integral from *t* to t+Δt can be removed from the outer integral,

(53) $$\begin{array}{cc} \Delta_{m,j}\left[t+\Delta t\right] & =\exp\left(-\int_{t}^{t+\Delta t}\frac{dt^{\prime \prime }}{\tau_{j}\left[t^{\prime \prime }\right]}\right) \end{array}$$

(54) $$\begin{array}{cc} & \times \left(\int_{t}^{t+\Delta t}\exp\left(-\int_{t^{\prime }}^{t}\frac{dt^{\prime \prime }}{\tau_{j}\left[t^{\prime \prime }\right]}\right)\frac{dT_{m}}{dt^{\prime }}dt^{\prime }+\int_{0}^{t}\exp\left(-\int_{t^{\prime }}^{t}\frac{dt^{\prime \prime }}{\tau_{j}\left[t^{\prime \prime }\right]}\right)\frac{dT_{m}}{dt^{\prime }}dt^{\prime }\right) \end{array}$$

The last term in Equation (52) is just $\Delta_{m,j}\left[t\right]$ and the first exponential term can be approximated by

(55) $$\exp\left(-\int_{t}^{t+\Delta t}\frac{dt^{\prime \prime }}{\tau_{j}\left[t^{\prime \prime }\right]}\right)\approx \exp\left(-\frac{\Delta t}{\tau_{j}\left[t\right]}\right)$$

so Equation (52) becomes

(56) $$\Delta_{m,j}\left[t+\Delta t\right]\approx \exp\left(-\frac{\Delta t}{\tau_{j}\left[t\right]}\right)\times \left(\int_{t}^{t+\Delta t}\exp\left(-\int_{t^{\prime }}^{t}\frac{dt^{\prime \prime }}{\tau_{j}\left[t^{\prime \prime }\right]}\right)\frac{dT_{m}}{dt^{\prime }}dt^{\prime }+\Delta_{m,j}\left[t\right]\right)$$

To evaluate the final integral in Equation (56) we note that $t^{\prime }$ only varies from *t* to t+Δt, so the inner integral can be approximated by

(57) $$\int_{t^{\prime }}^{t}\frac{dt^{\prime \prime }}{\tau_{j}\left[t^{\prime \prime }\right]}\approx \frac{t-t^{\prime }}{\tau_{j}\left[t^{\prime }\right]}$$

If the rate of temperature change is not very fast, the temperature derivative can be removed from the integral, so

(58) $$\begin{array}{cc} \int_{t}^{t+\Delta t}\exp\left(-\int_{t^{\prime }}^{t}\frac{dt^{\prime \prime }}{\tau_{j}\left[t^{\prime \prime }\right]}\right)\frac{dT_{m}}{dt^{\prime }}dt^{\prime } & \approx \frac{T_{m}\left[t+\Delta t\right]-T_{m}\left[t\right]}{\Delta t}\int_{t^{\prime }}^{t+\Delta t}\exp\left(-\frac{t-t^{\prime }}{\tau_{j}\left[t\right]}\right)dt^{\prime }\\ & \approx \left(\frac{T_{m}\left[t+\Delta t\right]-T_{m}\left[t\right]}{\Delta t}\right)\tau_{j}\left[t\right]\left(\exp\left(\frac{\Delta t}{\tau_{j}\left[t\right]}\right)-1\right) \end{array}$$

Expanding the integral in the last term, since exp(x)≈1+x, when *x* is small,

(59) $$\begin{array}{cc} \int_{t}^{t+\Delta t}\exp\left(-\int_{t^{\prime }}^{t}\frac{dt^{\prime \prime }}{\tau_{j}\left[t^{\prime \prime }\right]}\right)\frac{dT_{m}}{dt^{\prime }}dt^{\prime } & \approx \left(\frac{T_{m}\left[t+\Delta t\right]-T_{m}\left[t\right]}{\Delta t}\right)\tau_{j}\left[t\right]\left(\frac{\Delta t}{\tau_{j}\left[t\right]}\right) \end{array}$$

(60) $$\begin{array}{cc} & \approx T_{m}\left[t+\Delta \right]-T_{m}\left[t\right] \end{array}$$

Thus, we can find the value of $\Delta_{m}$ at t+Δt directly from its value at *t*, and Equation (56) can be approximated by

(61) $$\left.\Delta_{m,j}\left[t+\Delta t\right]\approx \exp\left(-\frac{\Delta t}{\tau_{j}\left[t\right]}\right)\left(T_{m}\left[t+\Delta t\right]-T_{m}\left[t\right]\right)+\Delta_{m,j}\left[t\right]\right)$$

The relaxation time, $\tau_{j}$, is dependent on the temperature of the patch, while the thermal strains in the patch and the wall are dependent on their respective local temperatures. The stress can therefore be calculated from:

(62) $$\sigma_{p}\left[t\right]\approx \left(\frac{E_{p}}{1-\nu_{p}}\right)\left(\psi_{0}\left(\epsilon_{f,s}-\epsilon_{f,p}\right)+\left(1-\psi_{0}\right)\sum_{j=1}^{N}w_{j}\left(\alpha_{s}\Delta_{s,j}\left[T_{s}\left[t\right]\right]-\alpha_{p}\Delta_{p,j}\left[T_{p}\left[t\right]\right]\right)\right)$$
