# Supplementary material for: Thermo-Mechanical Compatibility of Viscoelastic Mortars for Stone Repair
Source: Materials (Basel). 2016 Jan 18;9(1):56. doi: 10.3390/ma9010056 (PMC5456567; doi:10.3390/ma9010056)
Supplement: Supplementary file 1 [file materials-09-00056-s001.pdf]

Supplementary Materials: Thermo-Mechanical Compatibility of Viscoelastic Mortars for Stone Repair

Thibault Demoulin, George W. Scherer, Fred Girardet and Robert J. Flatt

1. Stress Relaxation of the Artificial Stone at Different Temperatures

The viscoelasticity of the acrylic-based repair mortar is investigated through the measurement of the relaxation of a load at a constant strain. This is done at several temperatures from −5 to 25 °C.

The stress relaxation is shown for every temperature in the following graphs. The fit of the data, performed through the NonLinearModelFit algorithm of *Mathematica* 10.2, provides E_0, the elastic modulus of the artificial stone, τ, the relaxation time, β, the exponent of the stretched exponential relaxation function, and ψ_0 the residual fraction of the stress that does not relax. These parameters are reported on the graphs of Figure S1.

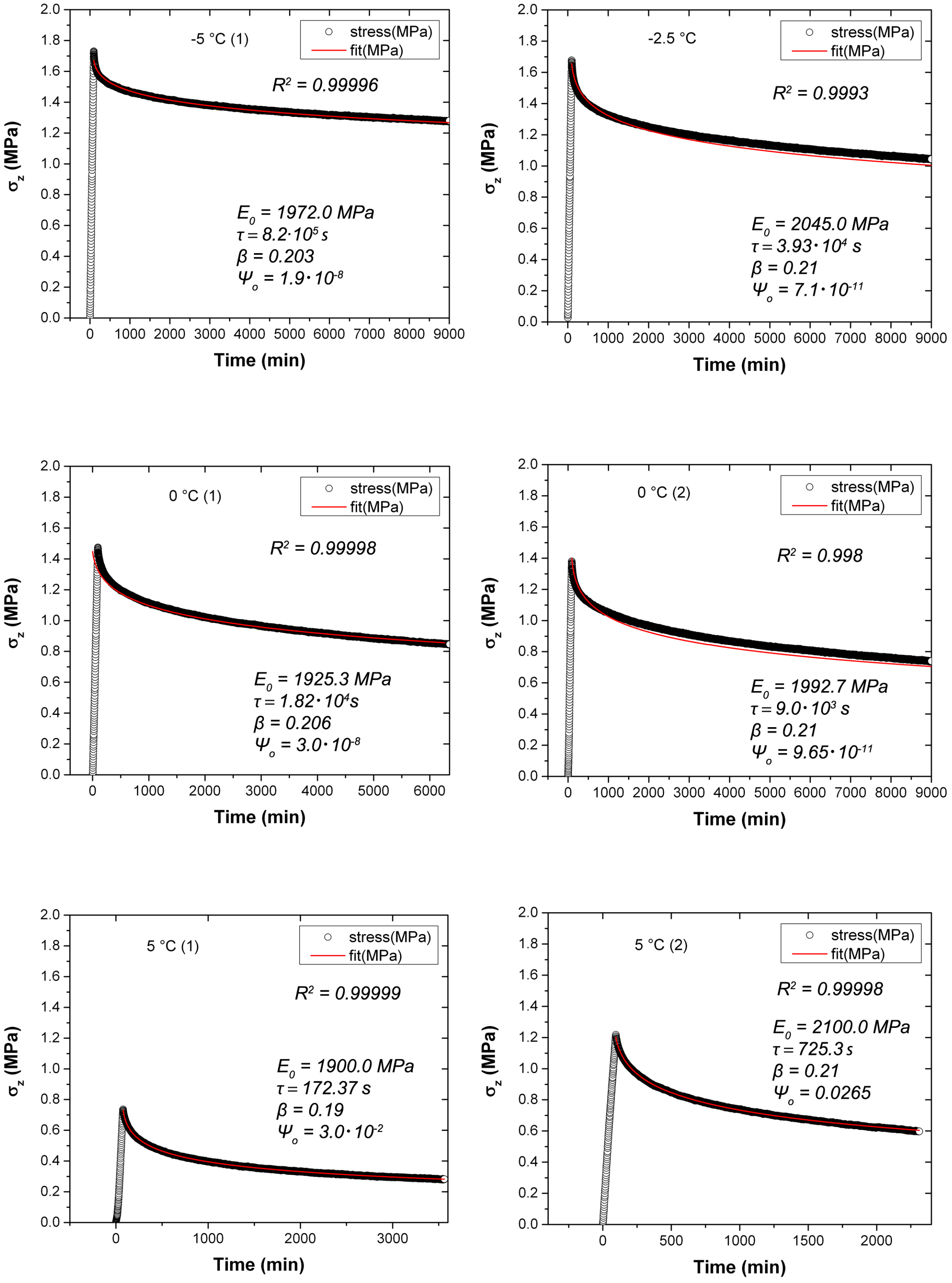

Figure S1. *Cont.*

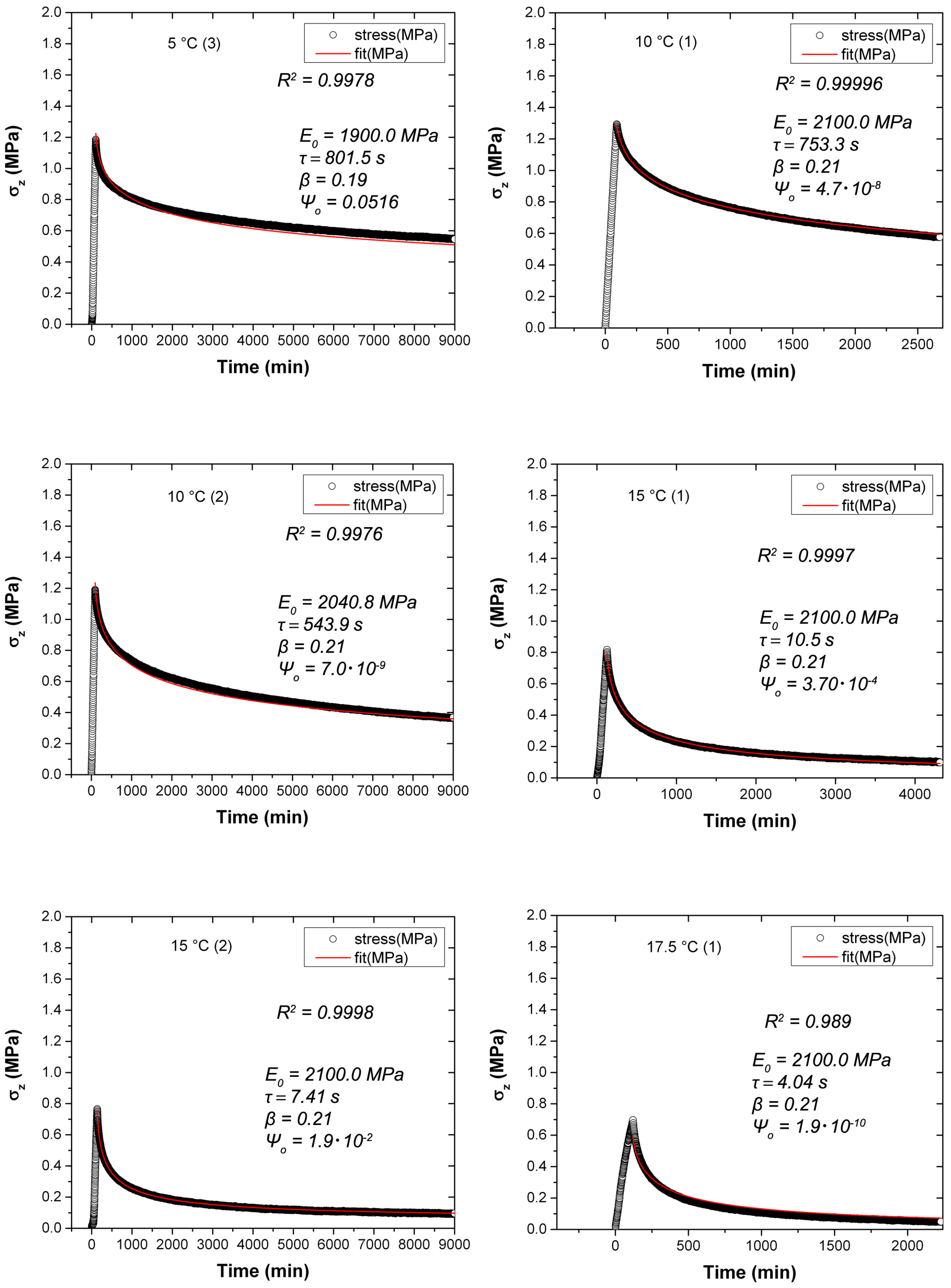

Figure S1. *Cont.*

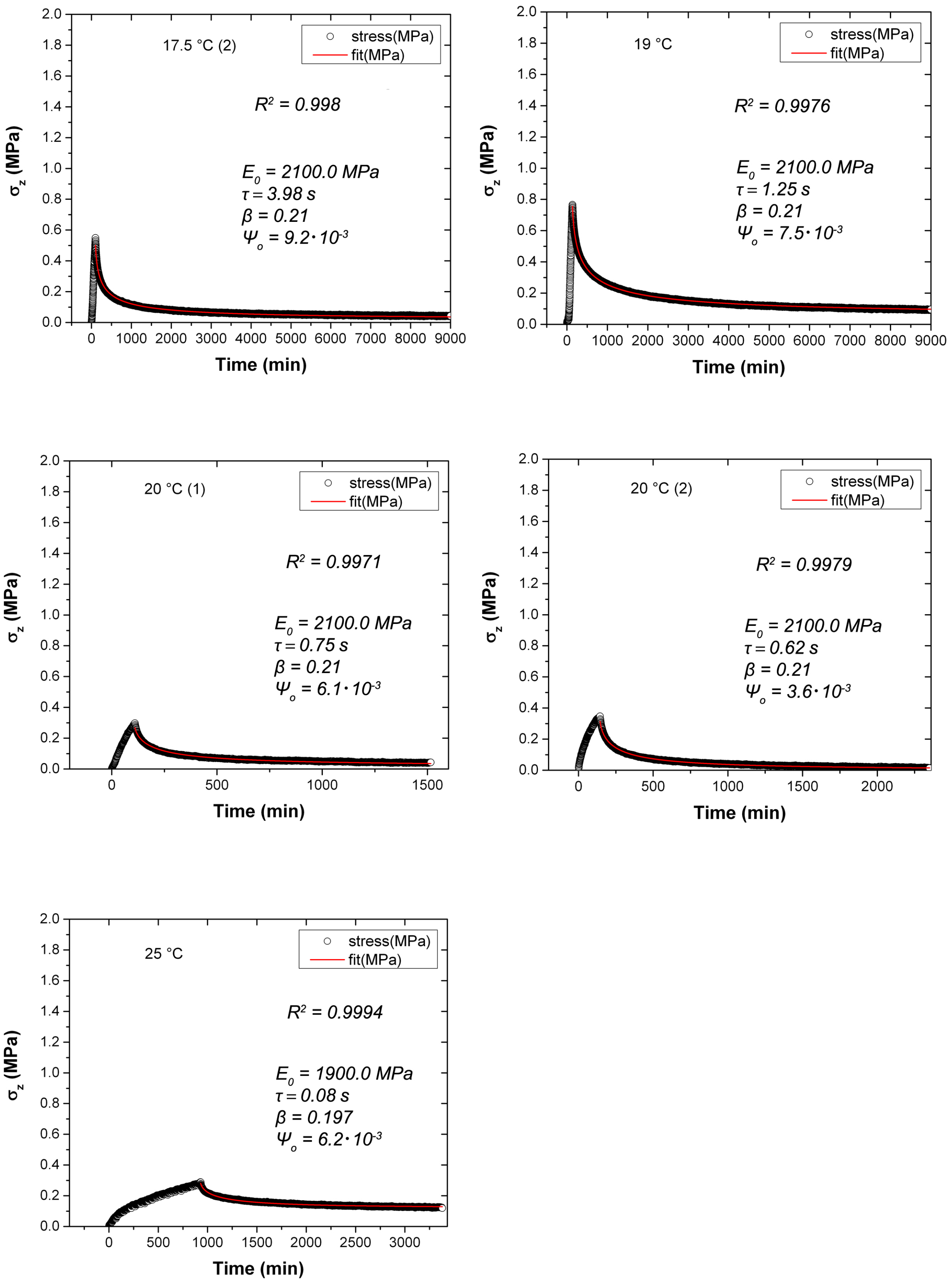

Figure S1. Stress relaxation curves at different temperatures. The temperature can be found on the top left of each graph, together with the run number at this temperature in parenthesis. Only the part of the stress obtained at a constant strain is fitted, and the parameters of the fit are reported on each graph.

2. Statistical Significance Calculation

The elastic modulus of the artificial stone seems to decrease with the temperature in the proportion of 4.2 MPa/°C in the range of 0 to 30 °C (see section Results, "Evolution of the elastic modulus with the temperature"). However, the uncertainty on the measurement is large and we need to assess whether this trend is significant or not. This is done by using a statistical test of which the theoretical fundations can be found in (Kendall and Stuart(1976)) and which has been successfully used in (Flatt(1999)). Its output is the probability that a parameter (here, the temperature) has an effect on a value (in our case, the elastic modulus), knowing the experimental error on each measurement.

The calculations are done using a Visual Basic script in Excel through the definition of a function that we call "tendency", which code is given below:

```
Function tendency(x, y, y_std_dev, n, nb_ev_pars_per_series)
'check how many values are given
nb_series = Application.CountA(x)
'create a dimension
for 1/sigma^2 ReDim one_over_sigma2(nb_series),
xx(nb_series), xy(nb_series)
'Calculate the sums necessary for the statistical treatment
s = 0
Sx = 0
Sy = 0
Sxx = 0
Sxy = 0
For bcl1 = 1 To nb_series
sigma_2 = y_std_dev(bcl1) ^ 2
s = s + 1 / sigma_2 Sx = Sx + x(bcl1) / sigma_2
Sy = Sy + y(bcl1) / sigma_2
Sxx = Sxx + x(bcl1) ^ 2 / sigma_2
Sxy = Sxy + x(bcl1) * y(bcl1) / sigma_2
Next bcl1
Delta = s * Sxx - Sx ^ 2
ordinate = (Sxx * Sy - Sx * Sxy) / Delta
variance_ordinate = Sxx / Delta
Slope = (s * Sxy - Sx * Sy) / Delta
variance_slope = s / Delta
covariance = -Sx / Delta
correlation_coefficient = -Sx / (s * Sxx) ^ 0.5
slope_over_error = Abs(Slope) / variance_slope ^ 0.5
degrees_of_liberty = Application.Sum(n) - nb_ev_pars_per_series * nb_series - 2
tendency = 1 - Application.TDist(slope_over_error, 1degrees_of_liberty, 1)
End Function
```

3. Mathematica Code for the Calculation of the Thermal Stresses

The calculations of the thermal stresses are done in *Mathematica* 10.2 using the following script. The Mathematica file (StressCalculation.nb) and the input data (Temperature_patch.dat and Temperature_stone.dat) are also included as supplementary materials.

Stress Development during a Thermal Cycle

Calculation of the stress using a sum of exponential terms

$$\sigma_{\mathrm{VE}}[T_k] \approx -\left(\frac{E_p}{1-\nu_p}\right)\left(\Psi_0\left(\epsilon_{f,s}-\epsilon_{f,p}\right)+(1-\Psi_0)\,\Delta[k]\right)$$

where

$$\Delta[k] = \sum_{j=1}^{N} w_j\left(\alpha_p\,\Delta_p - \alpha_s\,\Delta_s\right)$$

Prescribe the parameters: b = 0.206, N = 20

```
ClearAll["Global`*"]
```

In this section, you simply enter the values for N = Ntot and b = Exponentb and evaluate the function wfcns

```
Ntot = 20

Exponentb = 0.206
```

Create the functions:

```
Clear[τmin, τmax, b, tau, n, ntot, eq, w, eqs, i, appr, param]
```

The function τmin is τ_1/τ:

```
τmin[b_] := (0.0157 Exp[-7.93 b])^(1/b)
```

The function τmax is τ_N/τ:

```
τmax[b_] := (10.34 - 10.14 b)^(1/b)
```

Prescribe all the τ_n/τ as a function of b and ntot = N

```
tau[n_, b_, ntot_] := Exp[Log[τmin[b]] + ((n - 1)/(ntot - 1)) Log[τmax[b]/τmin[b]]]
```

Create an equation for the w_n by setting $t = \tau_i$:

```
eq[i_, b_, ntot_] :=
  0 == Exp[-(tau[i, b, ntot])^b] - Sum[w[n] Exp[-(tau[i, b, ntot]/tau[n, b, ntot])], {n, 1, ntot}]
```

This function creates a table with the N equations for a given b:

```
eqs[b_, ntot_] := Table[eq[i, b, ntot], {i, 1, ntot}]
```

This function creates a table with the names of the coefficients that we seek:

```
ans[ntot_] := Table[w[i], {i, 1, ntot}]
```

This function solves the equations and assigns the results to the w[n] = w_n

```
wfcns[b_, ntot_] := (dum = NSolve[eqs[b, ntot], ans[ntot]][[1]];
   Do[w[i] = dum[[i, 2]], {i, 1, ntot}])
```

This function is the approximation to the stretched exponential, where $\theta = t/\tau$:

```
appr[b_, ntot_, θ_] := Sum[w[n] Exp[-(θ/tau[n, b, ntot])], {n, 1, ntot}]
```

This is the original stretched exponential function:

```
ψ[θ_] := Exp[-θ^Exponentb]
```

Evaluate the weighting functions

```
wfcns[Exponentb, Ntot]
```

The approximation to the stretched exponential is now given by the function appr[Exponentb,Ntot,*θ*]

Plot the results

Plot the stretched exponential function for this value of *b*:

```
plorig = (PlotTitle = {"Stretched Exponential with b =", Exponentb};
  LogLinearPlot[ψ[θ], {θ, 10^-6, 10^2}, AxesLabel → {"Log10[t/τ]", "ψexact"},
   PlotStyle → {Black, Thick},
   BaseStyle → {FontFamily → "Arial", FontSize → 14, FontWeight → "Bold",
     Background → White}, Background → White,
   PlotLabel → TableForm[PlotTitle, TableDirections -> {Row, Row}]])
```

Plot the approximation:

```
plfit = (PlotTitle = {"Approximation to b =", Exponentb};
  LogLinearPlot[appr[Exponentb, Ntot, θ], {θ, 10^-6, 10^2},
   AxesLabel → {"Log10[t/τ]", "ψexact"}, PlotStyle → {Red, Dashed, Thick},
   BaseStyle → {FontFamily → "Arial", FontSize → 14, FontWeight → "Bold",
     Background → White}, Background → White,
   PlotLabel → TableForm[PlotTitle, TableDirections -> {Row, Row}]])
```

The fit is indistinguishable from the original function:

```
Show[plorig, plfit]

param = Table[{Log[10, tau[n, Exponentb, Ntot]], w[n]}, {n, 1, 20}];
```

This is the spectrum of relaxation times:

```
(PlotTitle = {"Spectrum for b =", Exponentb};
 ListPlot[param, Joined → True, AxesLabel → {"Log10[τn/τ]", "wn"},
  PlotStyle → {Red, Thick}, PlotRange → All,
  BaseStyle → {FontFamily → "Arial", FontSize → 14, FontWeight → "Bold"},
  PlotLabel → TableForm[PlotTitle, TableDirections -> {Row, Row}]])
```

These are the weighting factors, w_j

```
Table[w[i], {i, Ntot}]

Sum[w[i], {i, Ntot}]
```

These are the ratios of $\tau_0 / \tau_{0\,j}$

$$\lambda_j = \frac{\tau_0}{\tau_{0\,j}} \tag{5}$$

```
Clear[λ]

Table[λ[i] = 1 / tau[i, 0.3, Ntot], {i, Ntot}]

Sum[w[i] / λ[i], {i, Ntot}]
```

Importing the data.

```
SetDirectory["path"]

rawdatastone = Import["Temperature_stone.dat", "Table"][[3 ;;]];

rawdatapatch = Import["Temperature_patch.dat", "Table"][[3 ;;]];
```

The data consist in a date tag and a temperature value. The following line aims at reading properly the date tag.

For the stone:

```
datastone =
  rawdatastone /. {d_String, t_String, z__} :>
    {DateList[{d <> " " <> t, {"Day", ".", "Month", ".", "Year", " ", "Hour",
        ":", "Minute"}}], z};
nmaxs = Length[datastone]

datastone[[nmaxs, 1]]
```

For the patch (artificial stone):

```
datapatch =
  rawdatapatch /. {d_String, t_String, z__} :>
    {DateList[{d <> " " <> t, {"Day", ".", "Month", ".", "Year", " ", "Hour",
        ":", "Minute"}}], z};

nmaxp = Length[datapatch]

datapatch[[nmaxp, 1]]
```

To perform the calculation, the date is transformed into a time starting at zero, in seconds:

For the stone:

```
Do[times[i] = AbsoluteTime[datastone[[i, 1]]] - AbsoluteTime[datastone[[1, 1]]],
 {i, nmaxs}]

AbsoluteTime[datastone[[nmaxs, 1]]] - AbsoluteTime[datastone[[1, 1]]]

Do[dtimes[i] = times[i] - times[i - 1], {i, 2, nmaxs}]

dtimes[1] = dtimes[2]
```

For the patch:

```
Do[timep[i] = AbsoluteTime[datapatch[[i, 1]]] - AbsoluteTime[datapatch[[1, 1]]],
 {i, nmaxp}]

AbsoluteTime[datapatch[[nmaxp, 1]]] - AbsoluteTime[datapatch[[1, 1]]]

AbsoluteTime[datapatch[[1, 1]]]

Do[dtimep[i] = timep[i] - timep[i - 1], {i, 2, nmaxp}]

dtimep[1] = dtimep[2]

dtimep[9]
```

Creation of the temperature table

For the stone:

```
Do[Temps[i] = 273. + datastone[[i, 2]], {i, 1, nmaxs}]

Temps[1]

Temps[nmaxs]

Do[dTemps[i] = Temps[i] - Temps[i - 1], {i, 2, nmaxs}]

dTemps[1] = dTemps[2]

dTemps[1]

Tempsdata = Table[{times[i], Temps[i]}, {i, 1, nmaxs}];

plTdatas = ListPlot[Tempsdata, PlotStyle → Blue, Joined → True];
```

For the patch:

```
Do[Tempp[i] = 273. + datapatch[[i, 2]], {i, 1, nmaxp}]

Tempp[1]

Tempp[nmaxp]

Do[dTempp[i] = Tempp[i] - Tempp[i - 1], {i, 2, nmaxp}]

dTempp[1] = dTempp[2]

dTempp[1]

Temppdata = Table[{timep[i], Tempp[i]}, {i, 1, nmaxp}];

plTdatap = ListPlot[Temppdata, PlotStyle → Red, Joined → True];

dTemppdata = Table[{timep[i], dTempp[i]}, {i, 1, nmaxp}];

ListPlot[dTemppdata, PlotStyle -> Red, Joined -> True];
```

Creation of the temperature function

For the stone:

```
fcnTs = Interpolation[Tempsdata, InterpolationOrder → 1]

plTsfit = Plot[fcnTs[t], {t, times[1], times[nmaxs]}, AxesLabel → {"t (s)", "T (°C)"},
   PlotStyle → Blue, PlotRange → All];
```

For the patch:

```
fcnTp = Interpolation[Temppdata, InterpolationOrder → 1]

plTpfit = Plot[fcnTp[t], {t, timep[1], timep[nmaxp]}, AxesLabel → {"t (s)", "T (°C)"},
   PlotStyle → Red, PlotRange → All];
```

Use a time interval of 30 seconds:

```
dt = 30.

kmax = IntegerPart[times[nmaxs] / dt]
```

Physical parameters of the materials:

For the stone:

```
αs = 12.9 × 10^-6
```

For the patch:

```
Ep = 2003.

αp = 32.7 × 10^-6

νp = 0.4
```

Use the viscoelastic parameters estimated from the relaxation experiments:

```
τ0 = 4.87 × 10^-59

lnτ0 = Log[τ0]

Q = 39 350.
```

Part of the stresses that do not relax:

```
Ψ0 = 6.95 × 10^-3
```

Create an array of values for $T_{p,k}$ and $\Delta T_{p,k}$ and $T_{s,k}$ and $\Delta T_{s,k}$

For the stone:

```
t[0] = 0.

Ts[0] = fcnTs[0.]

fcnTs[10 000]

Do[{t[k] = dt k, Ts[k] = fcnTs[t[k]], dTs[k] = Ts[k] - Ts[k - 1]}, {k, kmax}]

Tsdata = Table[{t[k], Ts[k]}, {k, 0, kmax}];

ListPlot[Tsdata]
```

```
dTsdata = Table[{t[k], Ts[k] - Ts[0]}, {k, 0, kmax}];

plTsdata = ListPlot[dTsdata]
```

For the patch:

```
Tp[0] = fcnTp[0.]

fcnTp[10 000]

Do[{t[k] = dt k, Tp[k] = fcnTp[t[k]], dTp[k] = Tp[k] - Tp[k - 1]}, {k, kmax}]

Tpdata = Table[{t[k], Tp[k]}, {k, 0, kmax}];

dTpdata = Table[{t[k], Tp[k] - Tp[0]}, {k, 0, kmax}];
```

Computation of the strain mismatch for the calculation of the stresses that do not relax

```
Do[{eps[k] = αs * dTs[k] - αp * dTp[k]}, {k, kmax}]
```

Creation of the $\Delta_{m,j}$

For the stone:

```
Clear[ints, rat]

Do[ints[j, 0] = 0., {j, Ntot}];

Do[Do[ints[j, k] = Chop[(dTs[k] + ints[j, k - 1]) Exp[- λ[j] dt / (Exp[lnτ0 + Q / Tp[k - 1]])]],
    {j, Ntot}], {k, kmax}];

Clear[Δs]

Do[Δs[k] = Sum[w[j] ints[j, k], {j, Ntot}], {k, kmax}]

Δs[1400]

ΔTsvsT = Table[{Ts[k], Δs[k]}, {k, kmax}];

ΔTsvst = Table[{t[k], Δs[k]}, {k, kmax}];
```

For the patch:

```
Clear[intp, rat]

Do[intp[j, 0] = 0., {j, Ntot}];

Do[Do[intp[j, k] = Chop[(dTp[k] + intp[j, k - 1]) Exp[- λ[j] dt / (Exp[lnτ0 + Q / Tp[k - 1]])]],
    {j, Ntot}], {k, kmax}];

Clear[Δp]

Do[Δp[k] = Sum[w[j] intp[j, k], {j, Ntot}], {k, kmax}]
```

```
Δp[1400]

ΔTpvsT = Table[{Tp[k], Δp[k]}, {k, kmax}];

ΔTpvst = Table[{t[k], Δp[k]}, {k, kmax}];
```

Computation of Δ_m

```
Do[Δ[k] = Sum[w[j] (αs * Δs[k] - αp * Δp[k]), {j, Ntot}], {k, kmax}];
```

And finally the viscoelastic stresses σvst at a certain time can be computed, with the date as x-axis

The viscoelastic stresses are plotted against the time:

```
σvst =
  Table[{DateString[AbsoluteTime[datastone[[1, 1]]] + t[k],
     {"Year", "-", "Month", "-", "Day", "-", "Hour", ":", "Minute",
      ":", "Second"}], Ep/(1 - νp) * (Ψ0 * (eps[k]) + (1 - Ψ0) * Δ[k])}, {k, kmax}];

plσv = DateListPlot[σvst, PlotRange → All, AxesLabel → {"Date", "σ (MPa)"}]
```

The elastic stresses can be computed for comparison:

```
Clear[est, estress]

estress =
  Table[{DateString[AbsoluteTime[datastone[[1, 1]]] + times[i],
     {"Year", "-", "Month", "-", "Day", "-", "Hour", ":", "Minute",
      ":", "Second"}],
    est[i] = -Ep/(1 - νp) (αp (Tempp[i] - Tempp[1]) - αs (Temps[i] - Temps[1]))},
   {i, 1, nmaxp}];

plσe = DateListPlot[estress, Joined → True, PlotRange → All,
  AxesLabel → {"t (s)", "σ elastic (MPa)"}]
```

The elastic and viscoelastic stresses are plotted together against the date:

```
Show[plσe, plσv, PlotRange → All]
```

References

1. Kendall, M.G.; Stuart, A. *The Advanced Theory of Statistics. Vol. 1: Distribution Theory*, 2nd ed.; Griffin: London, UK, 1976.
2. Flatt, R.J. Interparticle Forces and Superplasticizers in Cement Suspensions. Ph.D. Thesis, Ecole polytechnique Fédérale de Lausanne, Lausanne, Switzerland, 29 September 1999.
